# Quantifying heterogeneous responses of fish community size structure using novel combined statistical techniques

**DOI:** 10.1111/gcb.13190

**Published:** 2016-02-15

**Authors:** Abigail M. Marshall, Grant R. Bigg, Sonja M. van Leeuwen, John K. Pinnegar, Hua‐Liang Wei, Thomas J. Webb, Julia L. Blanchard

**Affiliations:** ^1^ Department of Animal and Plant SciencesUniversity of SheffieldSheffieldUK; ^2^ Centre for EnvironmentFisheries and Aquaculture ScienceLowestoftUK; ^3^ Department of GeographyUniversity of SheffieldSheffieldUK; ^4^ Department of Automatic Control and Systems EngineeringUniversity of SheffieldSheffieldUK; ^5^ Institute of Marine and Antarctic StudiesUniversity of TasmaniaHobartAustralia

**Keywords:** biophysics, body size, climate change, demersal fisheries, empirical orthogonal functions, marine environmental change, NARMAX modelling, North Sea, size‐based indicators, spatial heterogeneity

## Abstract

To understand changes in ecosystems, the appropriate scale at which to study them must be determined. Large marine ecosystems (LMEs) cover thousands of square kilometres and are a useful classification scheme for ecosystem monitoring and assessment. However, averaging across LMEs may obscure intricate dynamics within. The purpose of this study is to mathematically determine local and regional patterns of ecological change within an LME using empirical orthogonal functions (EOFs). After using EOFs to define regions with distinct patterns of change, a statistical model originating from control theory is applied (Nonlinear AutoRegressive Moving Average with eXogenous input – NARMAX) to assess potential drivers of change within these regions. We have selected spatial data sets (0.5° latitude × 1°longitude) of fish abundance from North Sea fisheries research surveys (spanning 1980–2008) as well as of temperature, oxygen, net primary production and a fishing pressure proxy, to which we apply the EOF and NARMAX methods. Two regions showed significant changes since 1980: the central North Sea displayed a decrease in community size structure which the NARMAX model suggested was linked to changes in fishing; and the Norwegian trench region displayed an increase in community size structure which, as indicated by NARMAX results, was primarily linked to changes in sea‐bottom temperature. These regions were compared to an area of no change along the eastern Scottish coast where the model determined the community size structure was most strongly associated to net primary production. This study highlights the multifaceted effects of environmental change and fishing pressures in different regions of the North Sea. Furthermore, by highlighting this spatial heterogeneity in community size structure change, important local spatial dynamics are often overlooked when the North Sea is considered as a broad‐scale, homogeneous ecosystem (as normally is the case within the political Marine Strategy Framework Directive).

## Introduction

Determining the appropriate spatial scale for monitoring ecological communities has been cited as one of the most important challenges in applied ecology (Johnson, 2009). This challenge arises from the nonuniform response of species to their biotic and abiotic surroundings, many of which exist on different scales (Levin & Paine, 1974; Levin, 1992). One approach to determine spatiotemporal patterns is by identifying areas of predicted rapid change, known as ‘hot spots’, and to project expected ecological changes in these areas based on known physiological and community dynamics (Hannah *et al*., 2002; Belkin, 2009; Hobday & Pecl, 2014). An alternative approach is to quantify past, local ecological changes and attribute potential global, regional or local drivers to the corresponding changes. However, there is uncertainty surrounding the spatial scale at which to do this. For marine systems the global large marine ecosystem (LME; Sherman, 1991) classification scheme may provide a suitable scale for such analyses, especially when one considers the climate patterns at even larger scales that influence LMEs (Gherardi *et al*., 2010). However, there can be considerable heterogeneity in both environmental and anthropogenic drivers within LMEs. For instance, the North Sea LME has a number of thermal oceanic fronts influencing density, currents and nutrients (Belkin *et al*., 2009) that influence the marine ecosystem (Olson *et al*., 1994; Van Leeuwen *et al*., 2015). Additionally, fishing effort, a huge driver of ecosystem change (Jennings & Kaiser, 1998; Jennings & Blanchard, 2004), is not homogeneous across the North Sea (Jennings *et al*., 1999). Despite this, the North Sea LME is often characterized as a single cohesive ecosystem (EC, 2008; Greenstreet *et al*., 2011). With abundant ecological data available, methods to appropriately quantify heterogeneous change and thus manage ecosystems must be reviewed as systems adapt under a changing climate (Chave, 2013).

Many marine ecosystems are size structured, where lots of small individuals and fewer large individuals coexist (Sheldon *et al*., 1972), making body size of individuals a frequently employed proxy for ecosystem health and stability (Shin *et al*., 2005; Woodward *et al*., 2005; Greenstreet & Rogers, 2006; EC, 2008). Furthermore, trophic structure, a range of life‐history parameters and biological rates correlate strongly with body size (Sheldon *et al*., 1972; Blueweiss *et al*., 1978; Calder, 1984; Gillooly *et al*., 2001; Brown *et al*., 2004; Savage *et al*., 2004; Kingsolver & Huey, 2008; Rall *et al*., 2012; Reuman *et al*., 2014). The multifaceted effects of climate and fishing are known to disrupt the size structure of marine communities (e.g. Rice & Gislason, 1996; Blanchard *et al*., 2005). Body size is thus an important indicator of size‐structured, community‐level properties used in policy (EC, 2008) and for an ecosystem approach to fisheries management (Jennings & Dulvy, 2005).

Explicit size‐based indicators (SBIs) describe the distribution of body size. Commonly used examples include the large fish indicator (LFI), mean maximum weight, length ($\bar{W_{\max}}$, $\bar{L_{\max}}$), size spectrum slope and mean maturation size (Nicholson & Jennings, 2004; Shin *et al*., 2005). European policy, in the form of the Marine Strategy Framework Directive, calls for the use of biomass and the proportion of large fish (by weight) as indicators for targets in defining good environmental status of food webs (EC, 2010; Rogers *et al*., 2010). $\bar{W_{\max}}$, LFI and the size spectrum slope have all been used in the evaluation of management and targets in the North Sea (Nicholson & Jennings, 2004; Blanchard *et al*., 2014; Thorpe *et al*., 2015). We therefore use these three in our study.

In addition to using an indicator to describe the state of an ecosystem, such as body size distribution, drivers of ecological change also need to be examined. Environmental drivers (Daufresne *et al*., 2009; Cheung *et al*., 2012; Gale *et al*., 2013; Baudron *et al*., 2014), fishing pressure (Rice & Gislason, 1996; Grift *et al*., 2003; Jennings & Blanchard, 2004) and the interaction between them (Blanchard *et al*., 2005; Genner *et al*., 2010; Planque *et al*., 2010; Engelhard *et al*., 2014) have been found to cause changes in marine body size distributions.

Changes of the body size distribution in fish communities can be driven both directly and indirectly by increased seawater temperature (Perry *et al*., 2005; Daufresne *et al*., 2009; Gardner *et al*., 2011; Sheridan & Bickford, 2011). Direct effects can cause a reduction in body size distributions by (1) causing individuals to grow faster to a smaller size (Atkinson, 1994, 1995) although it is not fully understood why (Angilletta & Dunham, 2003; Atkinson *et al*., 2006) and (2) causing a shift in the population's distribution to deeper, cooler waters (Perry *et al*., 2005; Dulvy *et al*., 2008; Cheung *et al*., 2012; Pinsky *et al*., 2013) due to poorer recruitment success in warmer than average waters (Clark *et al*., 2003; Rindorf & Lewy, 2006; Rijnsdorp *et al*., 2009) and changing migration patterns (Nye *et al*., 2009). Indirect consequences of warmer waters on body size can occur through lower oxygen saturation levels (causing a constraint on fish growth and thus size, see Pörtner & Knust, 2007; Pauly, 2010; Cheung *et al*., 2012; Baudron *et al*., 2014), and phytoplankton changes (causing a mismatch in timing for food sources and recruitment, see Edwards & Richardson, 2004; Barnes *et al*., 2011). While temperature effects have been well explored, studies of oxygen effects are less numerous. They appear to influence physiology under lethal (Schurmann & Steffensen, 1997; Nilsson & Östlund‐Nilsson, 2004; Wu *et al*., 2003) and nonlethal conditions (Kinne & Kinne, 1962; Chabot & Dutil, 1999; Chabot & Claireaux, 2008). A reduction of body size can also occur by the removal of large individuals through size‐selective fishing (Jennings & Blanchard, 2004; Andersen & Pedersen, 2010) that also causes an increase in small individuals due to reduced predation pressure (Daan *et al*., 2005) and evolutionary adaptations (Rowell, 1993; Law, 2000; Grift *et al*., 2003; Olsen *et al*., 2004).

All these drivers are rarely distributed homogeneously in space. The North Sea LME has undergone changes (Beaugrand, 2004; MCCIP, 2013) at different rates across the region yet remains classified as a single ecosystem in policy (EC, 2008; Greenstreet *et al*., 2011). A marked recent warming in southern areas (~1.15 °C over 20 years) is greater than that of northern areas (~0.6 °C over 20 years; Holt *et al*., 2012a). Primary production varies according to stratification, shelf exchange rates, and riverine and atmospheric inputs, all of which are changing (Heath & Beare, 2008; Holt *et al*., 2012b). Oxygen levels have decreased with regions becoming hypoxic (Queste *et al*., 2012; Bendtsen & Hansen, 2013). Fishing effort is distributed heterogeneously throughout the North Sea (Jennings *et al*., 1999; Mills *et al*., 2007; Stewart *et al*., 2010). It is therefore plausible, if not highly probable, to expect trends in body size distribution of North Sea demersal fish to also be heterogeneous in space.

Here, we develop a spatially explicit statistical framework for highlighting local ecological change and investigating potential drivers of these changes. We use the North Sea demersal fish community as a case study. Firstly empirical orthogonal functions (EOFs), which have recently been applied in a fisheries setting (Morfin *et al*., 2012; Saraux *et al*., 2014), are used as a tool for determining major areas of change in size‐based indicators (SBIs). Secondly, we extract SBIs from three regions showing different changes, along with the corresponding environmental and fishing conditions. Finally, using $\bar{W_{\max}}$ as a case study, we use Nonlinear AutoRegressive Moving Average with eXogenous input (NARMAX) modelling as an illustrative example to investigate the potential drivers of size‐based change. NARMAX has been used widely in the fields of engineering (Billings, 2013), neuroscience (Zhao *et al*., 2013) and recently glacial climate dynamics (Bigg *et al*., 2014; Zhao *et al*., 2016). However, it has yet to be applied to fisheries. Using a highly spatial data set covering 29 years, we are able to apply EOFs and NARMAX in unison, for the first time, to highlight areas of major change and investigate potential drivers. The objective of this study therefore is twofold: (1) to mathematically quantify regional responses in size‐based indicators and identify the potential drivers and (2) to introduce a new statistical tool in fisheries research to aid marine management. By highlighting areas of contrasting ecological change under different environmental and anthropogenic conditions, this combination of statistical methods provides increased awareness of multifaceted effects found across the North Sea fishery.

## Materials and methods

### Study area

**Figure 1:**
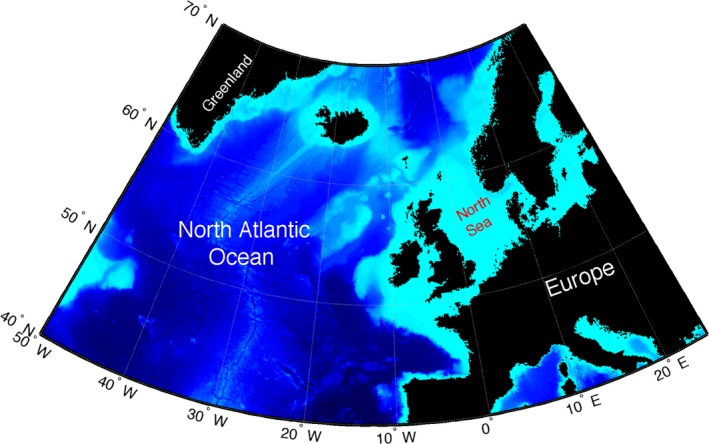
Map of the North Sea within the north‐west Atlantic Ocean and Europe. Blue colouring represents depth where dark blue is deep water; light blue is shallow.

Our study site is the North Sea (Fig. 1): this shelf sea is of international economic importance and undergoing very marked changes (see Introduction). We used a spatial resolution of 0.5° latitude × 1° longitude (ICES statistical rectangles) to conduct our analyses.

### Fish survey data

The North Sea International Bottom Trawl Survey (NS‐IBTS) quarter 1 data set is a collection of wintertime (January–March) fishery surveys taken annually from 1967 to present day in ICES statistical rectangles across the North Sea (downloaded from datras.ices.dk). The time period 1980–2008 was used here to ensure the greatest spatial coverage to allow for methodological consistency and confidence. Fish length was obtained for all demersal species caught during daytime GOV (Grande Ouverture Verticale) trawls. Individuals are identified to species level (if possible) and measured (cm). Data are converted to standardized units of catch per unit effort per length per haul (survey methodology and data processing are available at datras.ices.dk).

Fish lengths were converted to weight using conversion factors provided by Fung *et al*. (2012) and FishBase (Froese & Pauly, 2014), or the idealistic standard conversion factors: *a* = 0.01 and *b* = 3 (Cheung *et al*., 2012) if no weight‐length conversion factors existed. Only two of the total 159 taxa were converted using the latter process, *Micrenophrys lilljeborgii* (Norway bullhead) and any individuals labelled as ‘Cottidae’. Please see the supplementary materials for an overview of all species included in the analysis along with weight conversion values and quality control measurements (Daan, 2001).

### Size‐based indicators of fish community structure

The IBTS data set was used to calculate three size‐based indicators (SBIs) at the resolution of ICES statistical rectangles over the period 1980–2008: the large fish indicator (LFI), the mean maximum weight ($\bar{W_{\max}}$) and the size spectrum slope due to their use in North Sea policy and research (Rice & Gislason, 1996; Nicholson & Jennings, 2004; Shin *et al*., 2005; EC, 2008, 2013; Blanchard *et al*., 2014; Thorpe *et al*., 2015). Each SBI uses biomass (abundance multiplied by weight) as part of the main calculation – this biomass is the sum of each of the species’ biomass per haul. If more than one haul existed in an ICES statistical rectangle in 1 year, the biomasses were averaged across hauls.

The LFI is the ratio of the biomass of demersal fish >40 cm in length (*B*_*40*_) to the biomass of all demersal fish (*B*_*A*_):

(1) $$\text{LFI}=\frac{B_{40}}{B_{A}}$$

The second SBI used was the mean maximum weight ($\bar{W_{\max}}$) which calculates the maximum size relative to biomass across all species:

(2) $$\bar{W_{\max}}=\sum_{i}\frac{W_{\max,i}\cdot B_{i}}{B_{A}}$$

where *W*_max_ is the maximum observed weight of each species (*i*) found from the whole data set, *B*_*i*_ is biomass of species *i*, and *B*_*A*_ is the total biomass of all species. For the final SBI, the normalized biomass size spectrum slope, individuals were placed into size classes that conform to linear base two logarithms (e.g. 4–8 g wet weight, 8–16 g, 16–32 g…32768–65536 g) irrespective of species identity (Jennings & Blanchard, 2004). The total biomass of all individuals within each size class was calculated and divided by the bin width of the corresponding size class to give the normalized biomass. The size spectrum slope was determined from a linear regression slope of log_2_ (size class midpoint) against log_2_ (normalized biomass) from the point of the highest normalized biomass across the remaining larger weight classes. This was performed for each year in each rectangle.

Not all rectangles had data every year owing to occasional gaps in the geographical coverage of the surveys or to quality control procedures. In these cases, we interpolated SBIs using the mean value from all available adjacent rectangles (up to 8) for the specific year. Rectangles where fewer than 10 years of data existed were removed from our analyses after they had been used in the years they were present for, for greater interpolation accuracy. Consequently, we removed 11.83% of data from the LFI and $\bar{W_{\max}}$ leaving 164 rectangles. To have robust slope estimates, ICES rectangles were excluded where fewer than 7 of the 14 observed size classes were recorded. Of the 5394 ICES rectangle–year combinations (186 ICES rectangles, 29 years), 810 (15.02%) were excluded for this reason; 91.60% of which (i.e. 742) were rectangles that contained no data. After this, ICES rectangles with fewer than 10 years worth of data across the 29 years were also removed leaving 161 rectangles (of 186, so 13.44% removed altogether) in each year (4669 ICES rectangle–year combinations in total).

### Environmental data – GETM–ERSEM–BFM model

For a comprehensive cover of environmental data, variables were extracted from a 51‐year hindcast (1958–2008, although the first 20 years are considered ‘spin up’ time for the benthic system) of the validated coupled hydrodynamical–biogeochemical model GETM–ERSEM–BFM (Van der Molen *et al*., 2013; Van Leeuwen *et al*., 2013, 2015). The General Estuarine Transport Model (GETM, Burchard & Bolding, 2002; Stips *et al*., 2004; Burchard *et al*., 2014) is a fully 3D hydrodynamic, baroclinic, open source model coupled to ERSEM–BFM, the biogeochemical model developed jointly from the original ERSEM (European Regional Seas Ecosystem Model) and BFM (Biological Flux Model) codes by Cefas (UK) and NIOZ (the Netherlands) institutes. Further details can be found in the supplementary materials.

Spatially, the North Sea model set‐up of GETM–ERSEM–BFM covers the area 48.5–60.4°N, 5.66°W–16.20°E with a resolution of 0.1° × 0.167°. The northern and southern limits of the model are bounded by climatological averages which mean the environmental variables at these boundaries do not change annually. Therefore, to avoid this bias, we removed the areas close to these boundaries from our spatial grid. Monthly averages were extracted of variables that represent the demersal environment the species were most likely to experience: sea‐bottom temperature and sea‐bottom oxygen concentration. Depth‐integrated net primary production was taken as a proxy for food. For the purposes of our analysis, we averaged these extracted variables into ICES statistical rectangles to match the fish survey data.

### Fishing pressure

Data for fishing effort is restricted both spatially and temporally. The European Commission Scientific, Technical and Economic Committee for Fisheries (STECF), ICES and Jennings *et al*. (1999) have collected a variety of data although they are not compatible to generate a full time series at the ICES rectangle level. We carried out an analysis of the fishing effort and landings data available for the North Sea data to assess their limitations (see supplementary materials). Due to shortcomings of the fishing effort data, the next best method was to construct a multispecies proxy based on annual fishing mortality rates weighted by biomass of the target species (Daan *et al*., 2005). This was applied to each ICES statistical rectangle in each year as described below.

Fishing mortality rates (*F*) for stock‐assessed species are freely available from ICES (http://www.ices.dk/marine-data/dataset-collections/Pages/Fish-catch-and-stock-assessment.aspx). We used data from the following stock‐assessed demersal fish: *Gadus morhua* (Atlantic Cod), *Melanogrammus aeglefinus* (Haddock), *Pollachius virens* (Saithe), *Pleuronectes platessa* (Plaice), *Merlangius merlangus* (Whiting), *Solea solea* (Sole) and *Trisopterus esmarkii* (Norway pout). Together, this subsample of species makes up an average of 68% of the total biomass of all demersal species in the IBTS trawl surveys. While this means 32% of the biomass has not been represented, the remaining species are not target species thus their fishing mortalities are likely to be very low. Two species did not have *F* estimates for the full time period of our study available from ICES. Whiting assessments began in 1990; however, from 1990 to 2011, whiting *F* was highly correlated with haddock *F* (*r* ^2^ = 0.87) thus was extrapolated with a simple linear regression back to 1980. The same technique was used when Norway pout was extrapolated using saithe *F* (*r* ^2^ = 0.74) from 1980 to 1983. The equations used can be found in the supplementary materials.

Where biomass data did not exist, spatial interpolation was conducted as in the size‐based indicator section. To get a multispecies fishing mortality rate (*F*_*m*_) for each ICES rectangle, we weighted each species’ mortality by biomass:

(3) $$F_{m}=\sum_{i}\frac{B_{i}}{B_{\mathrm{SA}}}\cdot F_{i}$$

where *B*_*i*_ is the biomass of the species *i*,* B*_SA_ is the biomass of all the demersal stock‐assessed species, and *F*_*i*_ is fishing mortality for species *i*. This proxy for multispecies fishing mortality rate accounted for differences in the relative target species biomasses at the resolution of each ICES rectangle.

### Data analysis

#### Spatial heterogeneity of temporal change – empirical orthogonal functions

The purpose of the empirical orthogonal function (EOF) analysis was twofold: (1) to understand the spatial variation in temporal trends and (2) to highlight particular areas of interest that explain the maximum amount of variance. Mathematically, an EOF analysis is identical to principal component analysis. However, when used to define the spatiotemporal variation with spatially weighted data, the resulting functions are more commonly known as EOFs (Lorenz, 1956). Using a gridded latitude–longitude data set, the data were multiplied by $\sqrt{\cos\;(\text{latitude})}$ (Baldwin, 2001; Von Storch *et al*., 2004), where cos(latitude) represents the length of the parallel at the specified latitude relative to the length of the parallel at the equator. We performed a square‐root transformation because the data were subsequently used to create a covariance matrix for EOF analysis (the data were thus weighted by cos(latitude) as variance has a squaring term effectively nullifying the square‐root). Importantly, data sets being analysed by EOFs needs to be weighted if the geographical regions are not of equal area.

Once the data had been standardized (i.e. removal of the 29 year time‐averaged mean for each ICES statistical rectangle) and weighted spatially as described above, a covariance matrix was calculated. The data input was therefore a year x location grid (e.g. for the LFI: year = 29, location = 164 rectangles so a 29 × 164 grid). The covariance matrix is used to retrieve orthogonal predictors. Using singular value decomposition, a matrix algebraic method, eigenvectors and eigenvalues were calculated which were taken as our EOF modes and EOF principal components, respectively. The first mode of the SBIs was the only one extracted as this captures the pattern of greatest explained variance (here, ~28%). Additional EOF modes captured much less variance (<9%) making the first EOF mode the key pattern. The extracted mode therefore meets our first objective in the EOF analysis: quantifying the spatial variation in temporal trends.

To address the second objective, finding areas of interest, two extremes were chosen in terms of eigenvector values. An area of positive change was compared to an area of negative change and with an area of no change (of the respective SBI).

The average annual rate of change in SBIs and drivers were calculated by fitting a linear regression model at both the resolution of ICES statistical rectangles and for the North Sea as a whole. For environmental variables, monthly data were used that covered the same time period as the SBIs (1980–2008). A seasonal 30‐year average (1971–2001) was calculated and removed to normalize the data prior to computing the yearly averages that were subsequently used in the trend analysis. The estimated trends and associated spatial standard errors were used to test the significance of temporal change in SBIs and drivers across the North Sea. This was performed using the Mann–Kendall test where the null hypothesis was rejected at the 95% level.

#### Relative effects of drivers on size‐based change – NARMAX modelling

The use of the statistical model NARMAX (Nonlinear AutoRegressive Moving Average with eXogenous input) in our final stage of analysis simply provides a demonstration of one way in which to use the results obtained through the EOF analysis. The popularity of NARMAX stems in part from its ability to (1) identify linear and nonlinear relationships in data, (2) highlight quantitatively key explanatory variables that most strongly influence the dependent variable, (3) find the most likely relationship over a range of time lags and (4) highlight whether explanatory variables change in importance over time. Mathematical details of NARMAX are provided in the supplementary materials, and further methodological details can be found in Wei *et al*. (2010) and Billings (2013).

The formation of NARMAX is made up of model parameters (i.e. autoregressive, moving average and exogenous); a measured output (e.g. mean maximum weight); a noise term (which allows for error modelling, measurement errors and unmeasured disturbances to be accounted for); and explanatory variables which are taken as sea‐bottom temperature, sea‐bottom oxygen, depth integrated net primary production and fishing mortality, as well as their associated lags and all possible interactions between each individual and combined explanatory variable.

The number of model terms included in the initial full NARMAX model is based on the degree of nonlinearity and the combined number of variables for output, explanatory and error terms. This resulted in 84 potential model terms for this study. Running a model with this many variables is unrealistic, especially when so often in models and regressions, there are only a few significant model terms (regressors) which account for the greatest variance. NARMAX is powerful in determining which and how many model terms should exist in the final model using the forward regression orthogonal least squares algorithm (FROLS) (Chen *et al*., 1989; Wei *et al*., 2004, 2010). FROLS is efficient in model term selection and structure detection (including model validity test) under a nonlinear premise. In the most simplistic sense, the FROLS algorithm varies and tests each model term, and by comparing the corresponding output, each term is rated under the error reduction ratio (i.e. % variance change in the system when the individual model term is included – it is this metric that is used in our results). Terms that cause a statistically significant change in the output, even if small in variance influence, are always included in the final model. If any explanatory variables are identical or very closely correlated, NARMAX will only choose one of the variables to avoid colinearity. The FROLS algorithm thus forms a final parsimonious model (Wei & Billings, 2008; Wei *et al*., 2010).

The model validation is complex as many ‘standard’ methods used to validate models are based on linear systems and thus are not suitable in nonlinear models. Therefore, extended statistical validations (e.g. statistical correlation tests) and model predictive performance validation methods (e.g. model prediction output tests) were used to test the model following the protocol described in Chapter 5 of Billings (2013).

We used $\bar{W_{\max}}$ as the measured output. This output and the explanatory variables were extracted from each area of interest as identified by the EOF analysis. To reduce bias, we chose the same number of ICES rectangles (*I*_*n*_ = 8) in each area. The model estimation was conducted over 26 years (1980–2005), and the model test over the last 3 years (2006–2008), following a 90%/10% split which is the conventional practice for small sample modelling problems.

The strength of correlation between the model and empirical data was calculated using an r‐squared value. The r‐squared value ($r_{e}^{2}$) measures the ability of the model to characterize the trend in the output metric ($\bar{W_{\max}}$) by comparing the error amplitude to the observation amplitude (amplitude is also known as the norm of the error or observation) and thus is not the conventional r‐squared calculation (see supplementary material for full details). As the model test only ran for 3 years, calculation of the $r_{e}^{2}$ value was problematic, particularly when the amplitude of observations was close to zero. Therefore, we provide a Pearson correlation coefficient ($r_{p}^{2}$) for the final 3 years to indicate the linear relationship between the model and empirical data when $r_{e}^{2}$ was unable to provide a value for the model performance.

## Results

### Spatial variation in temporal trends of size‐based indicators

**Figure 2:**
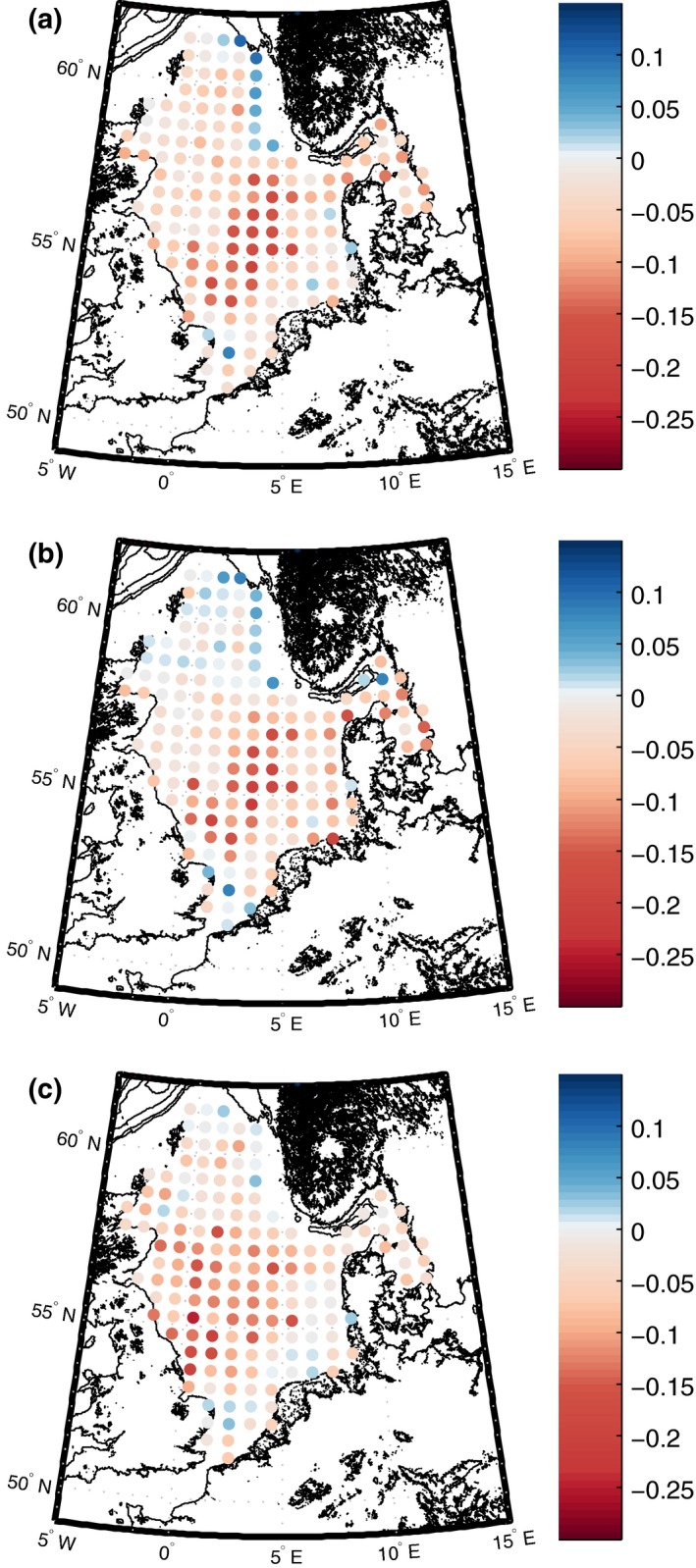
First mode from the empirical orthogonal function analysis of three size‐based indicators. This mode captures the main pattern of spatiotemporal variability. Modes are for (a) large fish indicator, (b) mean maximum weight and (c) size spectrum slope analysed from 1980 to 2008 using quarter 1 IBTS data set.

A significant trend in the North Sea LFI was identified as −0.0087 ± 0.0007 yr^−1^, equivalent to a reduction of 0.25 ± 0.021 between 1980 and 2008. Significant linear trends occurred in 96% of ICES statistical rectangles. Approximately 88% of ICES statistical rectangles showed a decline, with the remaining increasing. From the EOF analysis, the first mode accounted for 27.0% of the variance seen in Fig. 2(a). The central North Sea LFI showed the greatest decrease whereas the Norwegian trench region showed the greatest increase. Waters east of northern England and Scotland showed, on average, very little change. The Skagerrak and Kattegat exhibited a slight decrease in LFI, whereas the southern North Sea exhibited variable results.

$\bar{W_{\max}}$ showed the most prominent decline in size with a North Sea average annual change of −204 ± 21.5 gy^−1^ which equates to a reduction of 5916 ± 622 g between 1980 and 2008. Significant trends occurred across 48% of ICES statistical rectangles in the North Sea. The North Sea exhibited predominately a decrease in $\bar{W_{\max}}$ with 79% of ICES rectangles showing a decline over the 29 years. In terms of spatial changes through time, the first mode accounted for 28.3% of the variance and followed the pattern seen in Fig. 2(b). Similar to the LFI, the central North Sea exhibited the most substantial decline, whereas the Norwegian trench region witnessed the biggest increase in $\bar{W_{\max}}$ values, although changes were an order of magnitude lower than the central North Sea increase. Results for the southern North Sea, Skagerrak and Kattegat were consistent with LFI changes.

The trend of the normalized biomass size spectrum slope was −0.021 ± 0.0015 yr^−1^ and was significant when averaged over the entire region with 98% of ICES rectangles exhibiting significant temporal changes. Spatially, 86% of the ICES statistical rectangles showed a steepening of the size spectrum slope, with the remaining 14% showing an increase. The EOF first mode accounted for 26.6% of variance, the lowest of the three SBIs. This mode indicated decreased slope values (i.e. steepening of the slope) across large parts of the central North Sea, with increased slope values (i.e. slope becoming shallower) across north‐eastern parts of the North Sea (Fig. 2c). The southern North Sea showed patches of both increasing and decreasing slope values, but relatively low levels of change compared to the central North Sea.

To investigate the extremes in SBIs, three contrasting areas (‘areas of interest’) were defined such that, after the EOFs were performed, eigenvectors (X) for the mean maximum weight acted as boundaries for the following areas:

X < −0.166 (and <6°E) – central North Sea (negative change)

X > 0.01 – Norwegian trench region (positive change)

$\bar{X}$ ~ 0 (58.5–59°N, 2°W–0.5°E) – eastern Scottish coast (no change)

**Figure 3:**
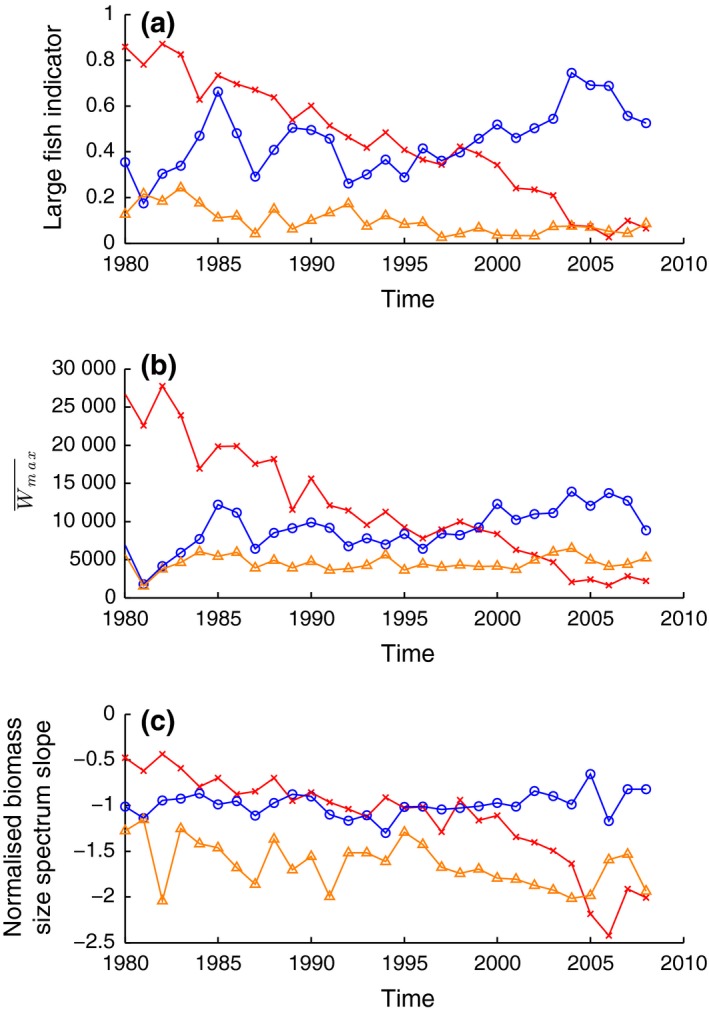
Time series of three size‐based indicators in the three ‘areas of interest’ defined by EOF mode 1. Time series in ‘area of interest’ 1 (cross, red, central North Sea), 2 (triangle, orange, eastern Scottish coast) and 3 (circles, blue, Norwegian trench region) as defined by the empirical orthogonal function analysis for (a) large fish indicator, (b) mean maximum weight (g) and (c) size spectrum slope. Calculated from 1980 to 2008 using quarter 1 IBTS data set.

The area of no change was taken as the eastern Scottish coast as the EOF analysis indicated this to be an area of no change (i.e. mean of X ~ 0). This particular area is of interest, as opposed to other areas of no change, because previous research (e.g. Perry *et al*., 2005; Pinsky *et al*., 2013) indicates communities are predicted to shift polewards due to climate change. The selected ‘no change’ region, however, has not shown this pattern. The boundaries of X were chosen to sample the same number of ICES rectangles (*I*_*n*_ = 8) from each region of change. Data from $\bar{W_{\max}}$ were extracted for NARMAX, and the LFI and normalized biomass size spectrum slope in the same region were extracted for a comparison (Fig. 3). The size spectrum slope was only averaged over seven ICES rectangles in the Norwegian trench region as rectangle 51F2 had been excluded for reasons explained in the methods section. Similarities between the LFI and $\bar{W_{\max}}$ compared to the size spectrum slope (Fig. 3) are most easily seen in the central North Sea (trends −0.029 ± 0.0008 y^−1^, −857 ± 29.41 gy^−1^, −0.053 ± 0.0033 y^−1^, respectively). The size spectrum slope, LFI and $\bar{W_{\max}}$ all indicate that the proportion of large fish declined from 1980 to 2008. Particularly from the late 1990s until 2005, the rate of decline of large fish proportionally was at its highest, picked up by all three indicators. In the Norwegian trench region, the size spectrum slope became slightly shallower throughout the time series (trend 0.004 ± 0.0018 y^−1^) despite more apparent increases in the LFI and $\bar{W_{\max}}$ (trends 0.009 ± 0.0023 yr^−1^ and 218.69 ± 47.75 gy^−1^, respectively). In fact, compared to the eastern Scottish coast (slope trend of −0.015 ± 0.0061 yr^−1^), the size spectrum in the Norwegian trench region was more steady. The LFI and $\bar{W_{\max}}$ showed an increase off the east Scottish coast (trend 17.51 ± 24.12 yr^−1^ and 0.0048 ± 0.0011 yr^−1^) although the trends were an order of magnitude lower compared to the other areas of interest. The smaller changes along the eastern Scottish coast compared to the Norwegian trench region and central North Sea were expected as this area was highlighted by the EOF analysis of $\bar{W_{\max}}$ to show little change over the 29‐year period.

### Environmental conditions and fishing in the three areas of interest

**Figure 4:**
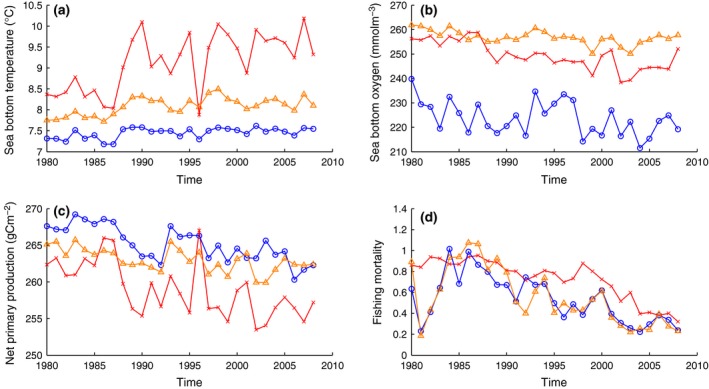
Time series of environmental and fishing data in the three ‘areas of interest’ defined by EOF mode 1. Time series over 1980–2008 in ‘area of interest’ 1 (cross, red, central North Sea), 2 (triangle, orange, eastern Scottish coast) and 3 (circles, blue, Norwegian trench region) as defined by the empirical orthogonal function analysis for (a) sea‐bottom temperature, (b) sea‐bottom oxygen, (c) depth integrated net primary production and (d) average multispecies fishing mortality rate. (a)–(c) calculated from the GETM–ERSEM–BFM output; (d) calculated from the IBTS quarter 1 data set and fishing mortalities from ICES.

Sea‐bottom temperature, sea‐bottom oxygen, depth integrated net primary production and relative fishing mortality were extracted in the three ‘areas of interest’ (Fig. 4) for both comparative purposes and NARMAX analysis. However, due to the northern boundary of the GETM–ERSEM–BFM model being at 60°N, if a rectangle from $\bar{W_{\max}}$ was at 60.5°N, the closest rectangle was taken instead for environmental data.

Significant increases in sea‐bottom temperature change occurred in 98.9% of the North Sea ICES rectangles with an average rise of 0.036 ± 0.001 °Cy^−1^ equating to 1.04 ± 0.03 °C increase over 29 years, in line with previous estimates (Holt *et al*., 2012a). In the central North Sea, eastern Scottish coast and Norwegian trench region, absolute sea‐bottom temperatures were an average of 9.14 ± 0.08 °C, 8.09 ± 0.06 °C and 7.45 ± 0.03 °C and over the 29‐year period trends were calculated as 0.05 ± 0.002 °Cy^−1^, 0.015 ± 0.003 °Cy^−1^ and 0.008 ± 0.004 °Cy^−1^, respectively.

A reduction in sea‐bottom oxygen was found across 87.6% of the North Sea. The average North Sea decrease in sea‐bottom oxygen was −0.26 ± 0.03 mmol m^−3^ yr^−1^ over 1980–2008, which is an overall decline of 7.62 ± 0.73 mmol m^−3^. The Norwegian trench region had much less oxygen available, an average of 223.7 ± 1.09 mmol m^−3^, compared to the central North Sea with 249.5 ± 0.54 mmol m^−3^, which itself was less than the eastern Scottish coast (256.8 ± 0.46 mmol m^−3^) on average between 1980 and 2008. However, the central North Sea showed a greater decrease in oxygen concentration of −0.50 ± 0.05 mmol m^−3 ^yr^−1^ compared to the Norwegian trench region of −0.36 ± 0.13 mmol m^−3 ^yr^−1^ and eastern Scottish coast of −0.18 ± 0.03 mmol m^−3^ yr^−1^.

Net primary production in the North Sea decreased in all areas, significantly so in over 94% of these areas. On average, net primary production decreased by −0.30 ± 0.02 gCm^−2 ^yr^−1^ equating to a 30‐year decrease of 8.66 ± 0.47 gCm^−2^. Regionally, the central North Sea average net primary production was 259.0 ± 0.27 gCm^−2^ from 1980 to 2008, compared to higher levels in the Norwegian trench region of 265.2 ± 0.35 gCm^−2^ and the eastern Scottish coast 263.0 ± 0.15 gCm^−2^. Furthermore, the central North Sea primary production decreased by −0.27 ± 0.01 g Cm^2 ^yr^−1^ compared to the Norwegian trench region decrease of −0.22 ± 0.06 g Cm^2 ^yr^−1^ and eastern Scottish coast decrease of −0.11 ± 0.02 g Cm^2 ^yr^−1^.

Over the course of 1980–2008, declines in the fishing mortality occurred in 96% of ICES rectangles. Across the North Sea, 93% of rectangles showed significant trends. At the North Sea scale, a trend of −0.018 ± 0.0005 yr^−1^ was significant. Fishing mortality was highest, on average, in the central North Sea being 0.73 ± 0.07. The eastern Scottish coast and Norwegian trench region were broadly similar with averages of 0.56 ± 0.1 and 0.54 ± 0.08, respectively. Fishing mortality in the central region, eastern Scottish region and the Norwegian trench region decreased at similar rates of −0.020 ± 0.002 yr^−1^, −0.021 ± 0.001 yr^−1^ and −0.016 ± 0.002 yr^−1^, respectively.

### Relative effects of size‐based change: implementing the NARMAX model

The NARMAX analysis showed that each area of interest was influenced by different environmental and fishing conditions used in the model. Changes in the central North Sea were highly associated with fishing of the current year and the previous year (96%), along with temperature change and the interactions between these two (Table 1). Oxygen and net primary production showed little association with the decrease in $\bar{W_{\max}}$ (<0.5%). The correlation between the model estimation and the raw data was very high ($r_{e}^{2}$ = 0.993 over 1980–2005). However, the amplitude of error in the original data compared to the model prediction was approximately the same making the $r_{e}^{2}$ value for 2006–2008 unfeasible (therefore $r_{p}^{2}$ = 0.54). The trends between 2006 and 2008 in the empirical data and model prediction are very similar which is encouraging. However, the absolute values for the model prediction underestimated the $\bar{W_{\max}}$ empirical data.

**Table 1:** NARMAX model results for ‘area of interest’ 1 (central North Sea), 2 (eastern Scottish coast) and 3 (Norwegian trench region). Index terms (Column 2) highlight order of importance of driving variable (i.e. environment/fishing) to the output (i.e. mean maximum weight). Model term column describes each variable where Temp = sea‐bottom temperature (°C), FM = fishing mortality, Netpp = depth integrated net primary production (gCm^−2^), Oxy = sea‐bottom oxygen (mmol^−3^). All are a function of *t* = time (years). Contribution column describes how much the model term contributes (%) to the change in mean maximum weight

Area of interest	Index	Model term	Contribution (%)
Central North Sea	1	FM(*t*) * FM(*t*−1)	95.68
	2	Temp(*t*−1) * FM(*t*−1)	1.49
	3	FM(*t*−2) * FM(*t*−4)	0.91
	4	Temp(*t*) * Temp(*t*−1)	0.23
	5	Oxy(*t*−4) * FM(*t*−1)	0.17
	6	Netpp(*t*−1) * FM(*t*−1)	0.27
Eastern Scottish coast	1	Netpp(*t*−3) * Netpp(*t*−3)	96.77
	2	Temp(*t*) * Oxy(*t*−1)	0.69
	3	Temp(*t*−2) * FM(*t*−2)	0.72
	4	Temp(*t*−2) * Oxy(*t*−3)	0.58
	5	Temp(*t*−2) * Netpp(*t*−4)	0.32
	6	FM(*t*−4) * FM(*t*−4)	0.06
Norwegian trench region	1	Temp(*t*−2) * Temp(*t*−2)	95.78
	2	Temp(*t*−2) * Oxy(*t*)	1.51
	3	Temp(*t*−4) * FM(*t*−4)	0.72
	4	Netpp(*t*) * FM(*t*−4)	0.65
	5	Temp(*t*−2) * FM(*t*)	0.19
	6	Netpp(*t*) * FM(*t*)	0.35

**Figure 5:**
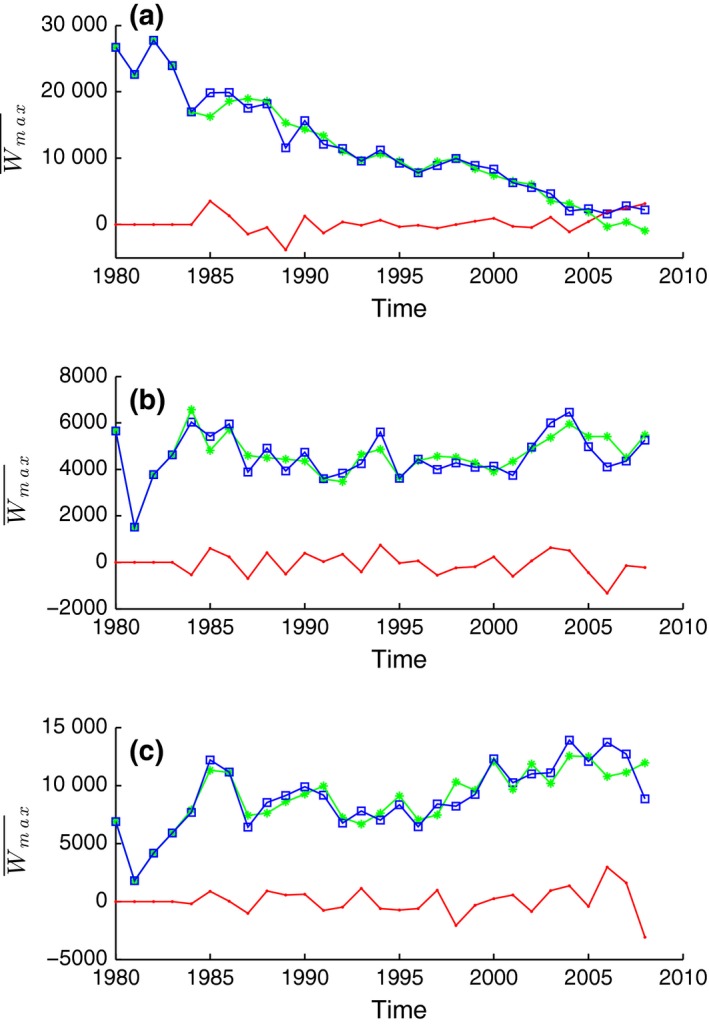
Results from the NARMAX model of the mean maximum weight. Mean maximum weight in (a) ‘area of interest’ 1 – central North Sea, (b) ‘area of interest’ 2 – eastern Scottish coast, and (c) ‘area of interest’ 3 – Norwegian trench region. Blue squares are the observed data from the IBTS, green stars are the modelled data using NARMAX, and red points are the difference between observation and prediction. The first 26 years (1980–2005) were used for model estimation; the final 3 years (2006–2008) were used for testing the model.

The NARMAX model suggested that the area of no change, the eastern Scottish coast, was strongly associated with net primary production (97%), with interactions of temperature, fishing and oxygen making up the final 3% (Table 1). The model estimation between 1980 and 2005 (Fig. 5b) was able to track the data with confidence ($r_{e}^{2}$ = 0.993). Despite a changing environment with no obvious changes in the community size structure, the model still had good predictive accuracy for the final 3 years ($r_{e}^{2}$ = 0.971), especially for 2007 and 2008.

In the Norwegian trench region, where $\bar{W_{\max}}$ increased, sea‐bottom temperature of 2 years prior was the main association (96%, Table 1). The remaining 4% was made up of the interactions of oxygen, fishing and temperature at different time lags. The correlation between the model estimation and the raw data was high ($r_{e}^{2}$ = 0.992 over 1980–2005). However, during the model prediction from 2006 to 2008, the model struggled to replicate the empirical data of $\bar{W_{\max}}$ (Fig. 5c) with a resulting lower error‐to‐signal ratio ($r_{e}^{2}$ = 0.951 over 2006–2008.

## Discussion

This study revealed substantial spatial heterogeneity around a 30‐year decline in community size structure for the North Sea large marine ecosystem. All three size‐based indicators (SBIs), which are widely used in research and policy, showed regions of distinct and opposing patterns at smaller spatial scales. The spatiotemporal heterogeneity was similar for the LFI and $\bar{W_{\max}}$ but differed slightly for the size spectrum slope. The combination of EOF analysis with NARMAX showed how identification of this spatial scale could help guide subsequent investigation of key drivers of change. To illustrate this, we focussed on $\bar{W_{\max}}$ as an indicator of community size structure and detected different patterns of change and potential drivers in the contrasting areas of interest. In the Norwegian trench region, increasing community size structure corresponded with increasing temperature, whereas decreasing size structure in the central North Sea was more strongly associated with changes in fishing mortality rates.

Ecosystems such as the North Sea are often studied as uniform systems for the purposes of regional assessment (Greenstreet & Rogers, 2006; Blanchard *et al*., 2012; Brotz *et al*., 2012; Fung *et al*., 2012). Other approaches use the biological and physical properties of the system itself to define appropriate subregions *a priori* (Zwanenburg *et al*., 2002; Van der Lingen *et al*., 2006; Wang *et al*., 2010; Van Leeuwen *et al*., 2015). Our approach instead directly mathematically quantifies the spatial structure in the temporal trends of ecological metrics. This allows interactions between drivers such as fishing and the environment to influence the system without any one driver defining its state that might consequently be used in defining a certain spatial area. The resulting high‐resolution outputs are especially appropriate for spatial management and policy. For instance, using this EOF–NARMAX framework, the spatial breakdown of regions could be used to inform where regulations are most likely to be effective for safeguarding important ecological measures such as community size structure. Although larger scale assessments for size‐based indicators, species biodiversity, abundance and trophic level are appropriate for reporting ecosystem states, the use of ecological indicators to support spatial management has thus far been limited. Our finding that trends and potential drivers of community size‐based indicators vary across the North Sea strongly advises against a ‘one‐size‐fits‐all’ approach to management, an approach which could simultaneously lead to both under‐ and overregulation in different areas, potentially impacting the well‐being of fishery and fishers.

The different conditions and trends in the environment and fishing pressure in the different regions alone are noteworthy. For example, compared to the Norwegian trench region, the central North Sea is warmer (with a greater trend of warming), shows oxygen decreasing at a faster rate, has lower primary production (with a greater decreasing trend) and has consistently higher fishing mortality rates. The eastern Scottish region is almost an environmental intermediate between these two areas, with only the sea‐bottom oxygen levels being the most favourable compared to each region. The fishing effort (hours fished) for both ICES divisions IVa (i.e. eastern Scottish coast and Norwegian trench region) and IVb (i.e. central North Sea) have decreased since 2000 although fishing in division IVb in absolute terms is far greater. These conditions point towards a more hostile environment for many demersal species across the North Sea, particularly in the central North Sea. Whether the positive (negative) change in the community size structure in the Norwegian trench region (central North Sea) associated with temperature (fishing) is an indirect (direct) effect reflecting of worsening habitat conditions in the central North Sea cannot be determined from this analysis. Changes in the community size structure can be altered from changes at the individual level (such as temperatures driving growth variability or human‐induced evolution) and/or the population level (through shifts or removal). The framework here is not able to definitively attribute mechanisms of any localized change but instead show where the changes are and the relevant strongest environmental/fishing association. Unfortunately, environmental changes in the Norwegian trench region are also tending towards less suitable conditions, though at a much slower rate. Despite the cold temperatures in this region, the oxygen concentration level is lower than the warmer central North Sea (Fig. 4a, b) due to warm, salty, oxygen‐deplete waters from the North Atlantic being forced into the deep trench. At this depth, waters cool but are unable to become oxygenated because of the year‐round saline stratification resulting from the much fresher Baltic outflow (which is less dense than the saltier North Sea and Atlantic waters). Therefore, if demersal species deepen, such as into the Norwegian trench region (Dulvy *et al*., 2008), the species would be driven out of the warmer areas into cool areas at a cost of reduced oxygen availability. However, a fish's metabolism reduces in cooler waters (Brown *et al*., 2004) so an analysis of scope for growth for each species could help determine the true cost (Claireaux & Lefrançois, 2007). This also highlights that warm waters do not always have less oxygen than cold waters, thus species are not always likely to be better off in cold waters on the assumption that these contain more oxygen. Concentrations are affected by spring blooms, currents, mixing, ventilation and water masses (Queste *et al*., 2012; Stendardo & Gruber, 2012), whereas oxygen solubility and saturation (i.e. the amount of oxygen the water is capable of containing) are bounded by temperature.

The spatial heterogeneity in drivers of the observed indicators of fish community structure revealed by the NARMAX analysis is in general agreement with previous findings. For instance, in the central North Sea, fishing pressure is known to be heavy (Jennings *et al*., 1999; Mills *et al*., 2007), highlighted both by fishing effort and landings data. The direct removal of individuals, and the resulting released predation pressure (Daan *et al*., 2005), has been reported as a primary driver of decreases in the abundance of large fish and community size structure (Jennings & Blanchard, 2004; Blanchard *et al*., 2005) with potential induced evolution occurring (Rowell, 1993; Law, 2000; Grift *et al*., 2003).

Differences in the temporal variability and changes in the size spectrum slope compared to LFI and $\bar{W_{\max}}$ may reflect differences in their intrinsic variability and sensitivity to drivers. Across the North Sea, the EOF first modes are comparable. However, when specific regions are extracted, the size spectrum slope appears to track changes consistently with the LFI and $\bar{W_{\max}}$ under fishing (i.e. central North Sea) compared to temperature changes (i.e. Norwegian trench region). The stronger response of the size spectrum to fishing compared to temperature has been suggested previously (Blanchard *et al*., 2005). This highlights the need to use a variety of size‐based indicators to understand changes to the community size structure that are likely to be influenced by both fishing and environmental effects. While modelling work has shown effects of fishing on the community size structure, environmental variability is often assumed to be part of the modelling error (Blanchard *et al*., 2014; Thorpe *et al*., 2015). Therefore, further work is required to understand how different mechanisms that structure body size distributions in communities respond to explicitly defined multiple stressors (e.g. environmental, fishing, chemical, pollution).

Fishing is known to have had wide‐ranging effects on North Sea fish abundance (Jennings & Blanchard, 2004). However, we were only able to use a proxy for fishing pressure because long‐term, continuous data of spatially resolved fishing effort across all North Sea fishing nations does not exist. Recent advances in the use of vehicle monitoring systems (Mills *et al*., 2007) and collections through the Scientific, Technical and Economic Committee for Fisheries (STECF) have brought together high‐resolution fishing effort across the North Sea, but due to different measurements of fishing effort (e.g. days fished, kilowatts days, fleet capacity) and countries recording data at different spatial resolutions, it is still currently impossible to compile a complete fishing effort time series. Jennings *et al*. (1999) showed how international collaboration can provide high‐resolution fishing effort while respecting fisherman confidentiality, and making such protocols routine practice would certainly improve our understanding of the true extent of fishing in the North Sea. Nevertheless, even with existing data, we believe that the EOF–NARMAX framework has proved effective in a fisheries context. The implementation of an EOF analysis results in several modes, each one describes how much variance a spatial pattern can explain. In this study, all EOF modes, other than the first, for each SBI explained <9% of the pattern variance thus we only considered the first EOF mode. However, it is always important when using EOF analysis to check modes beyond the first to determine whether the other modes need to be considered (i.e. ones of similar/large variance to the first mode). The use of North's rule of thumb can additionally determine whether the modes are statistically different (North *et al*., 1982). A caveat to the NARMAX modelling is that our measure of fishing mortality only represents 68% of the fish community biomass. However, the remaining 32% are not target species, and therefore, the fishing mortality is expected to be low. A final caveat is, both fishing mortality and the mean maximum weight use biomass in their calculation, potentially introducing a bias. Against this, however, we highlight 4 opposing arguments: (1) these two calculations are different and relative, not absolute under biomass; (2) NARMAX performs autocorrelation checks to ensure there is no bias; (3) if there was a bias, we would expect all areas of interest to be dominated by fishing, which was not the case; and (4) as outlined above, the fishing effort data we use, while imperfect, is the best available to our knowledge.

Fisheries provide an obvious application for the EOF–NARMAX framework, due to the existence of spatially extensive long‐term survey data, as well as a good understanding of potential drivers of community change. However, these statistical techniques can be of use in other ecological contexts where both suitable biological data (e.g. Loh *et al*., 2005; Cefas, 2014; McClatchie *et al*., 2014) and long environmental time series (e.g. Robock *et al*., 2000; Reynolds *et al*., 2002; Rayner *et al*., 2003; Gregg *et al*., 2005; Garcia *et al*., 2014a,b) are available. The use of kriging (Morfin *et al*., 2012) or specially formulated EOF algorithms (Taylor *et al*., 2013) allows for the use of EOFs in the presence of data gaps. NARMAX is powerful over long time periods (Bigg *et al*., 2014) as it has the ability to quantitatively determine the extent to which individual drivers are associated with changes, and when in time they dominate. It is recommended that the time series is not much shorter than 30 years to ensure the greatest accuracy from NARMAX. The EOF–NARMAX framework has great potential to spatially resolve past ecological changes and associations with potential drivers and to thus help predict how communities might respond to future global change scenarios.

As a specific case study of the utility of the EOF–NARMAX framework, in this analysis, we revealed heterogeneity in patterns of three key size‐based indicators of North Sea fish community size structure and subsequently showed an example of how one indicator was associated under multiple conditions. This enabled us to determine distinctive regions where community changes were strongly associated with temperature, depth integrated net primary production and fishing. These drivers are expected to change in the future (Jenkins *et al*., 2009; Collins *et al*., 2013; FAO, 2014), with possible negative consequences for dependent economic activities. Therefore the management of North Sea fisheries should take into account the multifaceted effects seen across different regions.

## Supporting information


**Data S1.** Data processing of the IBTS quarter 1 fisheries dataset, fishing mortality, landings, and fishing effort data.
