# Supplementary material for: Quantifying heterogeneous responses of fish community size structure using novel combined statistical techniques
Source: Glob Chang Biol. 2016 Feb 15;22(5):1755–68. doi: 10.1111/gcb.13190 (PMC4991301; doi:10.1111/gcb.13190)
Supplement: Supplementary file 1 — Data S1. Data processing of the IBTS quarter 1 fisheries dataset, fishing mortality, landings, and fishing effort data. [file GCB-22-1755-s001.docx]

Supplementary Materials

This supplementary material supports the paper ‘Quantifying Heterogeneous Responses of Fish Community Size Structure using Novel Combined Statistical Techniques’ by Marshall et al., 2016.

*Fish species*

All species recorded in the ICES North Sea International Bottom Trawl Survey were used and can be found in Table S1. In the cases where species have alternative names, we chose the same as Fung *et al*., (2012) but put in brackets other names. In accordance with the quality control assessment from Daan (2001) measurements that were less than 3 cm or greater than their species-specific Lmax (taken from [www.fishbase.org](http://www.fishbase.org)) were removed, the *Lepadogaster* record from 1991 and 1992, and the *Raja undulata* from 1990 were removed entirely and if *Leptoclinus maculatus* reached values greater than 20 cm, they were changed to *Lumpenus lumpretaeformis*. All pelagic species were removed.

*Fishing mortalities*

Between 1980 – 2011 fishing mortalities taken from ICES were used in calculations for a proxy for fishing. Only stock-assessed species have mortalities, therefore the following demersal species were used: *Gadus morhua* (Atlantic Cod), *Melanogrammus aeglefinus* (Haddock), *Pollachius virens* (Saithe), *Pleuronectes platessa* (Plaice), *Merlangius merlangus* (Whiting), *Solea solea* (Sole) and *Trisopterus esmarkii* (Norway pout). As these species make up ~68% of all biomass according to the IBTS dataset, we feel these are a sufficient representation of the demersal community. Values to 2011 were used to determine relationships for greater accuracy and can be found in Table S2.

Two species did not have fishing mortalities for the full time period of our study from ICES. Whiting assessments began in 1990. However from 1990 – 2011 whiting morality was highly correlated with haddock mortality with a simple linear regression explaining a large fraction of the variance (Figure S1, r^2^ = 0.87). Therefore from 1980 – 1989, the whiting mortality (*F_w_*) was extrapolated:

$$F_{W}=0.485F_{H}+0.081 (1)$$

where *F_H_* is the fishing mortality of haddock. The second species where the assessment did not extend to 1980 was Norway pout which began in 1983. Fishing mortality for Norway pout (*F_NP_*) between 1980 – 1982 was calculated based on a strong linear relationship (Figure S1, r^2^ = 0.74) found with fishing mortality of saithe (*F_S_*):

$$F_{NP}=1.8146F_{S}-0.203 (2)$$

*Fishing effort data*

We were unable to use fishing effort in our main analysis because the spatial resolution for the North Sea fishing countries was not available at the rectangle level throughout the time series. Instead datasets of different quality and resolution needed to be combined. Here we provide details of our amalgamation of North Sea fishing effort time series based on the finest spatial resolution possible, the ICES divisions IVa, IVb and IVc. The purpose is to illustrate the main challenges and limitations of the current ICES datasets for detailed spatio-temporal analysis.

The first time window for effort was calculated over 1980 – 1999 using data provided by Jennings *et al*., (1999). The original dataset had two components: (a) 1977 – 1995 effort from England, Scotland, Wales, Norway, and German otter and beam trawl fleets exist, and (b) 1990 – 1995 effort from these five countries with the addition of Denmark and Netherlands. All the data was aggregated thus individual country effort is unknown. The spatial patterns of 1990 – 1995 were very similar (see Figure 5 in Jennings *et al*., 1999). Therefore from 1980 – 1999 we assumed the same spatial pattern existed (i.e. *where* fishermen operated was the same each year). Practically this means we averaged each individual ICES statistical rectangle over 1990 - 1995 and assumed that the resulting spatial pattern was the same each year across 1980 - 1999. We took separate spatial pattern averages independently for otter trawls and beam trawls. As these seven countries have made up approximately 90% (±6%) of landings of otter and beam targeted species since 1977 (see next section), these countries are likely to capture the main North Sea fishing effort. We then amalgamated all the hours into ICES divisions IVa, IVb and IVc – this is the best resolution we could attain with the data we have available (for reasons described below). To impose annual variability of fishing (i.e. *how much* the fishermen fished) we related fishing effort to annual fishing mortality as this assumes lower fishing mortality is due to lower catch rates and thus less intensive fishing (vice versa under a high fishing mortality). Fishing mortalities used were those described in the above section. The species used were split into two groups: those most likely to be caught by otter trawls (cod, haddock, saithe, whiting and Norway pout) and those most likely to be caught by beam trawls (plaice and sole). To get an ‘average’ fishing mortality for beam (otter) trawls we simply averaged the species values within the beam-based (otter-based) mortalities together, to get two separate otter and beam trawl fishing mortalities over the last 29 years. The mortalities were standardised to the 1990 – 1995 mortality average. We proceeded to weight the 1980 – 1999 otter trawl spatial pattern by the otter fishing mortality; and the beam trawl spatial pattern by the beam fishing mortality. By this we mean each ICES statistical rectangle of effort each year was multiplied by the corresponding fishing mortality that was calculated for that year. The beam and otter trawls were added together to make an aggregate fishing effort distribution from 1980 – 1999. Distinguishing between the two trawls could be important, as they have not changed in unison over the last 30 years (i.e. compare Figure S2 with Figure S3).

The second time window considered was 2000 – 2002. From 2000 a major EU decommissioning project was implemented to reduce fleet size and capacity with around 25% of vessels being taken out of service from 2000 – 2011 (EC, 2013). Therefore we did not feel it was appropriate to use data from Jennings *et al*., (1999) to extend into the new millennium. Instead, using the North Sea effort from 1999 and the North Sea effort from 2003, we interpolated each ICES division (linearly for simplicity) to calculate the intermediate 2000, 2001 and 2002.

The third and final time window was 2003 – 2008. England and Wales, Scotland, Germany, Denmark and Netherlands otter and beam (regulated and unregulated) fleets effort were obtained from the STECF and amalgamated for ICES statistical rectangles and divisions IVa, IVb and IVc. First the English and Scottish fleets had to be readjusted. Data from England and Scotland is submitted to the STECF in days fished which is then multiplied by 24 to get hours fished. However, this is an unrealistic overestimation due to drive time and on-board processing (Engelhard *et al*., 2015). Therefore we use conversion factors that were calculated by Engelhard *et al*., (2015) who used days away from port and hours fished from English and Scottish government fisheries management datasets to calculate the conversion coefficients:

$$Hours fished\_EB=\frac{{FE}_{EB}}{24} . 7 (3)$$

$$Hours fished\_EO=\frac{{FE}_{EO}}{24} . 9.5 (4)$$

$$Hours fished\_SO=\frac{{FE}_{SO}}{24} . 10.6 (5)$$

where *FE_EB_*, *FE_EO_*, *FE_SO_* is the fishing effort provided by STECF for English beam trawlers, English otter trawlers, and Scottish otter trawlers, respectively. Equation 3 is the conversion for English beam trawlers, equation 4 for English otter trawlers, and equation 5 for Scottish otter trawlers, the *Hours fished* is the readjusted STECF fishing effort used for effort calculations.

As Norway is not part of the EU, it does not submit fishing effort data to STECF. However, as Norway is part of the compiled data in Jennings *et al*., (1999) it is crucial to have some representation of Norway in the later years. If not, a decline in fishing may simply be an artefact due to the exclusion of Norway in recent datasets, especially considering their high landing contribution from division IVa relative to other countries (~50% since the early 1970s). The only effort data available from Norway from 2002 onwards is for ICES subarea IV (i.e. the whole of the North Sea) for the mixed fisheries. In order to disaggregate the data to improve the resolution we made the assumption that ~95% of effort came from division IVa, ~5% from IVb, and <0.5% from IVc (varied year by year). This is based on landings data where Norway landed these proportions of cod, haddock, whiting, saithe, plaice, sole and turbot species between 2002 – 2008 in division IVa, IVb, and IVc. These species were chosen as they are aggregated as such by the ICES Mixed Fisheries working group (WGMIXFISH, 2014). The data were given in kW days and as the fleet size, engine size and power were not submitted, we were unable to amalgamate this series with the STECF data. We simply use this data to look at the relative changes over time. While we accept this is not an ideal approach, lacking spatially resolved effort data we were limited in our approach.

*Fisheries landings*

Landings data was downloaded from ICES Catch Statistics. Landings are in tonnes, the equivalent of live weight. Discarded catch and other non-landed quantities were not included. No alterations to the data had to be made based on any precautions.

We use the landings primarily to show: (a) 7 species are sufficient to represent the majority of fishing pressure targets, (b) 7 countries are sufficient to represent North Sea demersal fishing pressure, and (c) the spatial pattern of fishing effort over 1980–1999 must be based on 1990–1995 data.

Landings data are available from 1950 – 2010, however only data from 1977 – 2008 were used. Data from the required divisions were kept: IVa, IVb and IVc. These three regions make up landings caught from the North Sea (subarea IV) and were aggregated for this purpose. All countries that had landings in these areas were kept for comparative purposes. If no ICES region was specified they were removed. Only the stock-assessed demersal fish as mentioned above were used. Landing values which were recorded as ‘<0.5’ were put at 0.25. We accept these are not the exact values; the values are so small relative to complete landings (less than 0.0001%) that the influence on the total landings is negligible. We can also assume they balance out as 0.25 will be an overestimate for some, and an underestimate for others. The landings from 1989 onwards include England, Wales and Northern Ireland as an aggregate which we were unable to separate. Despite Jennings *et al*., 1999 not having Northern Ireland, we did not feel the inclusion likely to be a big influence. Landings from Northern Ireland in the North Sea only occurred in division IVa and the total landings from these region up until 1989 (i.e. before it became aggregated) was 106 tonnes, negligible compared to the total landings from the North Sea on an annual basis (<0.01%). Therefore it is unlikely that the inclusion of Northern Ireland as an aggregate will influence the use of landings comparisons.

Using fishing mortalities we split species into beam-based and otter-based. Though the beam trawl is likely to normally include a variety of other landed flatfish (e.g. brill, turbot, dab), plaice and sole have made up over 73% of flatfish landings since 1977 (Figure S2). In the mid 1980s this was around 84%, but due to a severe decrease in plaice landings in the early 1990s they accounted for 61% of flatfish where it continued to fluctuate between 61% and 69% until the end of our time series, 2008. For the otter trawl, we found the 5 specified species (cod, whiting, haddock, saithe, Norway pout - CWHSN) dominated with their combined landing weight often over 90% (Figure S3). Beam and otter trawls are dominated by these 7 species, making them a good representation of the fish community that are targeted by fishing pressure thus supporting (a).

From 1977 – 2008 in England, Scotland, Wales, Norway and Germany, landings of all flatfish species (vs plaice and sole only) accounted for 32.3% (vs 24.7%) of total annual beam-related landings relative to all countries that fish the North Sea. The inclusion of Denmark and Netherlands increase this to 91.0% (vs 91.2%). Therefore it is vital that Dutch and Danish fishing effort is included at all points in time. The otter related landings from 1977 – 2008 in England, Scotland, Wales, Norway and Germany for all species (vs cod, haddock, whiting, saithe, Norway pout only) account for 59.6% (59.6%) of total landings of otter trawl species likely to be caught relative to all countries that fish the North Sea. The inclusion of Denmark and Netherlands increases this to 89.3% (vs 89.4%) again highlighting the importance of including these two countries (Figures S2, S3). From landings we have shown that England, Scotland, Wales, Norway, Germany, Denmark and the Netherlands contribute over 89% of beam and otter landings, thus supporting statement (b).

Despite there being 30 countries that have fished in the North Sea since 1950, the locations where they fish are not all available. Therefore in order to create a time series at a high spatial resolution was limited by the data we have. As we were unable to tell where Denmark and Netherlands were fishing in 1980 – 1989, our best estimate is to assume they fished in the same places in the years that we do know (i.e. 1990 – 1995). In (b) we demonstrated that the use of 7 countries accounted for > 89% of landings from North Sea making it imperative to include Denmark and Netherlands. By default, therefore, (c) has effectively been supported. Furthermore, the Dutch fleet between 1975 and 1995 was made up of 500 to 600 ships (Daan, 1977) therefore the hours fished in 1990 – 1995 are likely to be similar to that of the whole time period 1975 – 1995 (Jennings *et al*., 1999). Exclusion of these fleets from 1980 – 1989 could prove to be a major oversight. As no other spatial data is available, and the inclusion of these two countries is vital, our only option is to use the spatial pattern generated from 1990 – 1995.

From the 7 countries that we took as a representative of North Sea demersal fishing, landings from beam trawls accounted for 91% of total beam-related landings (sole, plaice); otter trawls accounted for 89% of total otter-related landings (cod, haddock, whiting, saithe, Norway pout). Therefore these take into account the majority of the industrial demersal fishing in the North Sea and are likely to give a good representation of fishing effort through time.

The fishing effort showed differences between ICES divisions IVa, IVb and IVc (Figure S4), though the two divisions where an area of interest exists are IVa and IVb. Division IVa steadily declined until 1998 (despite a sharp increase in the early 1980s) with a small increase since. Hours fished from 2003 displayed a sharp decrease, which may coincide with EU fleet decommissioning, though it is somewhat sharper than has been recorded (EC, 2013). Effort in division IVb was an order of magnitude higher than in divisions IVa and IVc. Effort was steady from the early 1990s until a rise in 2000 but this is likely a mismatch between the different datasets used. Data from Norway, however, showed a substantial increase where effort tripled over a 6-year period. This increase was seen across the North Sea and while we have weighted it by landings, the relative change in all regions is the same. The missing Norway data that we were unable to convert to hours fished may account for some of the mismatch.

*Further details of NARMAX*

In the main paper we introduced a new to fisheries statistical model known as Nonlinear AutoRegressive Moving Average with eXogenous input (NARMAX) to fisheries science.

The most commonly used NARMAX form is a power-form polynomial represented as:

where y is the measured output, θ values are autoregressive, moving average and exogenous model parameters. The variable x is defined as:

$$x_{m}(t)=\left\{\begin{matrix} y(t-m)\text{ }1\leq m\leq n_{y} \\ u(t-(m-n_{y}))\text{ }n_{y}+1\leq m\leq n_{y}+n_{u} \\ e(t-(m-n_{y}-n_{u}))\text{ }n_{y}+n_{u}+1\leq m\leq n_{y}+n_{u}+n_{e} \end{matrix}\right.$$

where x is made up of 3 terms: the measured output (y); the explanatory variables (u); and a noise term (e) which allows modelling error, measurement errors/noise and unmeasured disturbances to be accounted for. The noise term is calculated by e(t) = y(t) – t(t|t-1) where y(t|t-1) is an instantaneously predicted value which an estimated model has produced. The output term (y), explanatory terms (u) and noise (e) all have associated maximum time lags (therefore in each term the amount of variables between no lag and the maximum lag equals n_y_, n_u_, n_e_, respectively). The number of model terms included in the initial NARMAX model (M) is based on the degree of non-linearity (L) and the combined number of variables for output, explanatory and error terms (n), and is calculated as:

$$M=\frac{\left( n+L \right)!}{[n!L!]}$$

where n = n_y_ + n_u_ + n_e_. This resulted in 84 potential model terms for this study. Running a model with this many variables is unrealistic. To determine the final number of model terms within NARMAX, forward regression orthogonal least squares (FROLS) algorithm (Chen *et al*, 1989; Wei *et al*, 2004; Wei *et al*., 2010) is implemented. The aim of FROLS is to find and include the least amount of terms that are able to account for the most variance; any additional terms have no statistically significant effects on the system variance. The FROLS algorithm is complex. Readers are directed to Wei & Billings (2008) and Wei *et al*., (2010) for a comprehensive detailing on the iterative algorithm which includes the code to instruct the FROLS algorithm. These papers, and references within, are more detailed in the formulation and derivation of NARMAX, NARX and explicit algorithms.

*R-squared values*

Let Y = [y(1), y(2), …, y(N)] *^T^* be a measured system response vector and E = [e(1), e(2), …, e(N)]*^T^* be an error vector whose elements are defined as e(t) = y(t) – y(t|t-1) (t =1,2,…, N) where y(t|t-1) is an instantaneously predicted value from the model. Therefore:

R2 = 1 - ||E||^2^/||Y||^2^

where the symbol ||x||^2^ indicates the norm of a vector x.

The reason why R2 is defined in such a way (rather than following its traditional definition R2 = 1 - ||E||^2^/||Y-mean(Y)||^2^) is that the modelling task here is to track and interpret the overall varying trend of the target signal using the given potential independent variables. In dynamical data modelling studies, the term ||E||^2^/||Y||^2^ is referred to as the error-to-signal ratio, which has a clear meaning when applying the NARMAX method to solve dynamical process modelling (Wei & Billings, 2008; Wei *et al*., 2010; Billings, 2013). The value R2 = 1 - ||E||^2^/||Y||^2^ provides an index to measure how well the model can follow and characterize the trend in the target signal y(t).

In cases where the data needs to be pre-processed (e.g. centralization and normalization etc), the traditional definition is normally considered. In our study here, we have used the raw data - one advantage of using raw data is that all the original physical meanings in the data can be reserved, and this can help interpret the resultant models.

*GETM-ERSEM-BFM*

The applied hydro-biogeochemical model was GETM-ERSEM-BFM. This model combines the GETM hydrodynamical code (a fully 3D baroclinic hydrodynamic model, see Burchard & Bolding, 2002; Stips *et al.*, 2004; Burchard *et al.*, 2014) with the ERSEM-BFM biogeochemical model.

The ERSEM model (European Regional Seas Ecosystem Model, see Baretta et al., 1995; Ruardij & Raaphorst, 1995; Ruardij *et al*., 1997; Ebenhöh *et al*., 1997) was developed in the 1990s to represent marine biogeochemical processes with the specific aim to model functional types (rather than species) and allow for internally varying nutrient ratios within its organisms. It incorporates four phytoplankton types (diatoms, flagellates, picophytoplankton, dinoflagellates), four zooplankton types (microzooplankton, heterotrophic nanoflagellates, omnivorous and carnivorous mesozooplankton), 5 benthic types (megabenthos, deposit feeders, filter feeders, meiobenthos, infaunal predators) and pelagic and benthic (aerobic and anaerobic) bacteria. The dynamic cycles for nitrogen, phosphorous, silicate, oxygen and carbon are included. The sediment is divided in three layers of varying depth: the oxic layer, denitrification layer and anoxic layer. Subsequent reprogramming of ERSEM in Fortran 95 led to the formation of the more modular BFM model (Biological Flux Model, see http://bfm-community.eu) in the early 21st century. This code was applied in oceanic form (Vichi *et al*., 2003, 2004, 2007) but also in shelf seas applications (Ruardij *et al*., 2005).

The ERSEM-BFM code applied here stems from further collaborative development by the Cefas and NIOZ institutes of the shelf seas BFM code: it therefore includes specific processes to represent shelf seas dynamics not found in ERSEM or BFM codes. Additional functional types include: small diatoms and *Phaeocystis* colonies in phytoplankton. Further additions include production of transparent exopolymer particles (TEP) by nutrient-stressed diatoms and *Phaeocystsis*, leading to macro-aggregate formation and increased sinking rates. A simple suspended particles matter (SPM) parameterisation, assuming proportionality to bed-shear stress induced by surface waves, has been included as described in Van der Molen *et al*. (2014) to improve representation of the underwater light climate. Improvements in benthic-pelagic coupling have led to a benthic module comprising 53 state variables, see Van der Molen *et al*. (2013) for more details including validation for benthic-pelagic exchange. For other applications of the ERSEM-BFM model see van Leeuwen *et al*. (2013, 2015).

The hindcast coverage, both spatially and temporally, was well suited to the rest of the data in this study with a coverage of 48.5-60.4°N, 5.66E-16.20°W with a resolution of 0.1° x 0.167°. The northern, southern and eastern limits of the model are bounded by climatological averages which means the environmental variables in these areas do not change annually. Therefore in order to avoid this static bias we removed these areas from our spatial grid. Temporally, the model runs from 1958 - 2008 with daily outputs. The first 20 years of this period were `spin up' time, in order to establish a steady state, particularly with respect to nutrient concentrations in the bed. Initial values were taken from a previous multi-decadal simulation.

**Figures**

**Figure S1**. Relationship between haddock fishing mortality and whiting fishing mortality in the North Sea (top), and saithe fishing mortality and Norway pout fishing mortality in the North Sea (bottom). Data used from ICES catch statistics.

**Figure S2**. North Sea landings (tonnes) including all species most likely caught by beam trawls from: all countries (red solid); England, Scotland, Wales, Norway, Germany, Denmark and Netherlands (red dashed); England, Scotland, Wales, Norway and Germany (red dots); and plaice and sole from: all countries (blue solid); England, Scotland, Wales, Norway, Germany, Denmark and Netherlands (blue dashed); England, Scotland, Wales, Norway and Germany (blue dots). Data from ICES catch statistics from 1977 - 2008.

**Figure S3**. North Sea landings (tonnes) including all species most likely caught by otter trawls from: all countries (red solid); England, Scotland, Wales, Norway, Germany, Denmark and Netherlands (red dashed); England, Scotland, Wales, Norway and Germany (red dots); and cod, whiting, haddock, Norway pout and saithe from: all countries (blue solid); England, Scotland, Wales, Norway, Germany, Denmark and Netherlands (blue dashed); England, Scotland, Wales, Norway and Germany (blue dots). Data from ICES catch statistics from 1977 - 2008.

**Figure S4**. Time series of fishing effort for ICES division (a) IVa, (b) IVb, (c) IVc and (d) subarea IV. Time series where the straight line is from Jennings *et al*., (1999) in hours fished, the single dots are interpolated points, the dotted line is data from the STECF in hours fished, and the dashed line is the Norwegian fishing effort in kW days in ICES division (a) IVa, (b) IVb and (c) IVc. All three divisions are shown in (d) with division IVa dashed (pink), IVb dotted (orange) and IVc a straight line (green); the dotted vertical line is where the Jennings *et al*., (1999) data stops, and the dashed vertical line is where the STECF data starts. Effort data comes from England and Wales, Scotland, Germany, Norway, Denmark and Netherlands.

**Tables**.

| Latin name | Common name | a | b |
| --- | --- | --- | --- |
| *Acentronura* | Pygmy pipehorse | 0.0002 | 3.2326 |
| *Agonus cataphractus* | Pogge | 0.0091 | 2.905 |
| *Amblyraja radiata* | Starry ray, Thorny skate | 0.0056 | 3.121 |
| *Anarhichadidae* | Wolffishes, sea wolves | 0.003 | 3.2491 |
| *Anarhichas lupus* | Wolffish, Catfish | 0.003 | 3.2491 |
| *Anarhichas minor* | Spotted wolffish | 0.003 | 3.2491 |
| *Anguilla anguilla* | European eel | 0.0006 | 3.313 |
| *Anguillidae* | Freshwater eels (spawn in seawater) | 0.0006 | 3.313 |
| *Arnoglossus* | Scaldfish | 0.0047 | 3.218 |
| *Arnoglossus imperialis* | Imperial scaldfish | 0.0028 | 3.34 |
| *Arnoglossus laterna* | Mediterranean scaldfish | 0.0065 | 3.096 |
| *Artediellus atlanticus* | Atlantic hookear sculpin | 0.0065 | 3.096 |
| *Blenniidae* | Combtooth blennies | 0.0093 | 3 |
| *Bothidae* | Lefteye flounders | 0.0047 | 3.218 |
| *Brosme brosme* | Tusk, cusk | 0.0051 | 3.198 |
| *Buglossidium* | Only one species exists in this genus | 0.0078 | 3.128 |
| *Buglossidium luteum* | Yellow sole, solenette | 0.0078 | 3.128 |
| *Callionymidae* | Dragonets | 0.0135 | 2.6857 |
| *Callionymus* | Dragonets | 0.0135 | 2.6857 |
| *Callionymus lyra* | Common dragonet | 0.0086 | 2.927 |
| *Callionymus maculatus* | Spotted dragonet | 0.0162 | 2.5781 |
| *Callionymus reticulatus* | Reticulated dragonet | 0.0158 | 2.552 |
| *Caproidae* | Boarfishes | 0.2218 | 1.9707 |
| *Capros aper* | Boar fish | 0.2218 | 1.9707 |
| *Chelidonichthys cuculus* | Red gurnard (Aspitrigla cuculus) | 0.0045 | 3.2228 |
| *Chelidonichthys lucerna* | Tub gurnard (Trigla lucerna) | 0.008 | 3.061 |
| *Chelidonichthys lucernus* | Tub gurnard misspelt | 0.008 | 3.061 |
| *Chimaera monstrosa* | Rabbit fish | 0.0003 | 3.475 |
| *Ciliata mustela* | Fivebeard rockling | 0.0064 | 3 |
| *Ciliata septentrionalis* | Northern rockling | 0.0055 | 3.1785 |
| *Conger conger* | Conger eel | 0.0002 | 3.509 |
| *Cottidae* | Sculpins | 0.01 | 3 |
| *Crystallogobius linearis* | Crystal goby | 0.008 | 3.1614 |
| *Cyclopteridae* | Lumpfishes, lumpsuckers | 0.0587 | 2.939 |
| *Cyclopterus lumpus* | Lumpsucker | 0.0587 | 2.939 |
| *Diplecogaster bimaculata* | Two-spotted clingfish | 0.0141 | 2.737 |
| *Dipturus batis* | Blue skate, grey skate, flapper skate | 0.0036 | 3.0787 |
| *Dipturus linteus* | Sailray, pale rale, white skate | 0.0036 | 3.0787 |
| *Echiichthys vipera* | Lesser weever fish (Trachinus vipera) | 0.0129 | 2.947 |
| *Echiodon drummondii* | Pearlfish | 0.059 | 3.1304 |
| *Enchelyopus cimbrius* | Fourbeard rockling (Rhinonemus cimbrius) | 0.0035 | 3.1062 |
| *Entelurus aequoreus* | Snake pipefish (Enterurus aequerius) | 0.0002 | 3 |
| *Etmopterus spinax* | Velvet belly latern shark | 0.0019 | 3.212 |
| *Eutrigla gurnardus* | Grey gurnard | 0.0034 | 3.26 |
| *Gadus morhua* | Atlantic cod | 0.0039 | 3.2434 |
| *Gaidropsarus* | Lotid fishe | 0.0072 | 2.865 |
| *Gaidropsarus macrophthalmus* | Big-eyed rockling | 0.0063 | 2.95 |
| *Gaidropsarus mediterraneus* | Shore rockling | 0.0034 | 3.098 |
| *Gaidropsarus vulgaris* | Three-bearded rockling | 0.012 | 2.547 |
| *Galeorhinus galeus* | School shark, tope shark | 0.0098 | 3.0085 |
| *Glyptocephalus cynoglossus* | Witch flounder | 0.0013 | 3.435 |
| *Gobiidae* | Goby | 0.008 | 3.1614 |
| *Gobius* | Goby | 0.0119 | 3.0495 |
| *Gobius auratus* | Golden goby | 0.0119 | 3.0495 |
| *Gobius cobitis* | Giant goby | 0.0113 | 3.128 |
| *Gobius niger* | Black goby | 0.0124 | 2.971 |
| *Helicolenus dactylopterus* | Blackbelly rosefish | 0.151 | 3.0456 |
| *Hippoglossoides platessoides* | Long rough dab | 0.007 | 2.978 |
| *Hippoglossus hippoglossus* | Atlantic halibut | 0.235 | 1.797 |
| *Lampetra fluviatilis* | River lamprey | 0.0011 | 3.141 |
| *Lepidorhombus whiffiagonis* | Megrim | 0.0134 | 2.746 |
| *Leptagonus decagonus* | Atlantic poacher | 0.0091 | 2.905 |
| *Leptoclinus maculatus* | Daubed shanny | 0.0244 | 2.0439 |
| *Lesueurigobius* | Goby | 0.0026 | 3.515 |
| *Lesueurigobius friesii* | Frie's goby | 0.0026 | 3.515 |
| *Leucoraja circularis* | Sandy ray | 0.0024 | 3.233 |
| *Leucoraja fullonica* | Shagreen ray (Raja fullonica) | 0.0024 | 3.233 |
| *Leucoraja lentiginosa* | Speckled skate | 0.0024 | 3.233 |
| *Leucoraja naevus* | Cuckoo ray (Raja naevus) | 0.0024 | 3.233 |
| *Limanda limanda* | Dab | 0.0071 | 3.119 |
| *Liparis* | Snailfish | 0.0207 | 2.9691 |
| *Liparis liparis* | Sea-snail | 0.0122 | 2.9892 |
| *Liparis montagui* | Montagu's sea-snail | 0.0292 | 2.949 |
| *Lophiidae* | Goosefish | 0.01 | 3.2 |
| *Lophius budegassa* | Black-bellied angler | 0.0044 | 3.345 |
| *Lophius piscatorius* | Angler fish, monkfish | 0.0166 | 2.9776 |
| *Lumpenus lampretaeformis* | Snakeblenny (Lumpenus lumpretaeformis) | 0.0244 | 2.0439 |
| *Lycenchelys sarsii* | Sar's wolfeel (Lycenchelys sarsi) | 0.0417 | 2.2532 |
| *Lycodes vahlii* | Vahl''s eelpout | 0.0417 | 2.2532 |
| *Melanogrammus aeglefinus* | Haddock | 0.0052 | 3.156 |
| *Merlangius merlangus* | Whiting | 0.0042 | 3.1842 |
| *Merlucciidae* | Merluccid hakes | 0.0036 | 3.1469 |
| *Merluccius merluccius* | (European) hake | 0.0036 | 3.1469 |
| *Micrenophrys lilljeborgii* | Norway bullhead | 0.01 | 3 |
| *Microchirus* | Soles (genus) | 0.008 | 3.141 |
| *Microchirus variegatus* | Thickback sole | 0.008 | 3.141 |
| *Microstomus kitt* | Lemon sole | 0.0042 | 3.2695 |
| *Molva dypterygia* | Blue ling | 0.0019 | 3.149 |
| *Molva molva* | Common/European ling | 0.001 | 3.4362 |
| *Mugilidae* | Mullet | 0.0107 | 3.0328 |
| *Mullus barbatus* | Red mullet | 0.0139 | 2.9087 |
| *Mullus surmuletus* | Striped red mullet, goatfish | 0.0101 | 3.0201 |
| *Mustelus* | Smooth-hounds | 0.0041 | 2.9185 |
| *Mustelus asterias* | Starry smooth-hound | 0.002 | 3.079 |
| *Mustelus mustelus* | Common smooth-hound | 0.0062 | 2.758 |
| *Myoxocephalus scorpioides* | Arctic sculpin | 0.0178 | 3.0378 |
| *Myoxocephalus scorpius* | Bull rout, Shorthorn sculpin | 0.0126 | 3.1235 |
| *Myxine glutinosa* | Hagfish | 0.0033 | 2.699 |
| *Nerophis ophidion* | Straighnose pipefish | 0.0004 | 3 |
| *Pagellus erythrinus* | Common pandora | 0.0171 | 2.906 |
| *Pegusa lascaris* | Sand sole | 0.007 | 3.13 |
| *Petromyzon marinus* | Sea lamprey | 0.0008 | 3.1956 |
| *Pholis gunnellus* | Butterfish, Rock gunnel | 0.0043 | 3.018 |
| *Phrynorhombus norvegicus* | Norwegian topknot (Zeugopterus noregicus) | 0.0078 | 3.1457 |
| *Phycis blennoides* | (Greater) fork-beard | 0.0022 | 3.3892 |
| *Platichthys flesus* | European flounder | 0.0087 | 3.0978 |
| *Pleuronectes platessa* | European plaice | 0.0078 | 3.0541 |
| *Pollachius pollachius* | Pollack | 0.0061 | 3.115 |
| *Pollachius virens* | Saithe | 0.0042 | 3.1753 |
| *Pomatoschistus* | Goby | 0.0068 | 3.0965 |
| *Pomatoschistus lozanoi* | Lozano's goby | 0.0068 | 3.0965 |
| *Pomatoschistus microps* | Common goby | 0.0068 | 3.0965 |
| *Pomatoschistus minutus* | Sandy goby | 0.0062 | 3.173 |
| *Pomatoschistus pictus* | Painted goby | 0.0073 | 3.02 |
| *Psetta maxima* | Turbot | 0.0046 | 3.3972 |
| *Raja* | Rays | 0.0035 | 3.1746 |
| *Raja brachyura* | Blonde ray | 0.0028 | 3.233 |
| *Raja clavata* | Thornback ray | 0.0032 | 3.194 |
| *Raja microocellata* | Smalleyed ray | 0.0049 | 3.117 |
| *Raja montagui* | Spotted ray | 0.0023 | 3.2051 |
| *Raja undulata* | Undulate ray | 0.0042 | 3.124 |
| *Rajidae* | Skates | 0.0036 | 3.1632 |
| *Raniceps raninus* | Tadpole fish | 0.0062 | 3.2667 |
| *Scophthalmus maximus* | Turbot | 0.0046 | 3.3972 |
| *Scophthalmus rhombus* | Brill | 0.0055 | 3.3047 |
| *Scorpaena scrofa* | Red scorpionfish | 0.0121 | 3.124 |
| *Scyliorhinus* | Catsharks | 0.0031 | 3.029 |
| *Scyliorhinus canicula* | Lesser spotted dogfish | 0.0031 | 3.029 |
| *Scyliorhinus stellaris* | Nursehound | 0.0031 | 3.029 |
| *Sebastes* | Rockfish | 0.0093 | 3.1585 |
| *Sebastes marinus* | Rose fish, ocean perch, redfish | 0.0071 | 3.18 |
| *Sebastes viviparus* | Norway redfish/haddock | 0.0115 | 3.1369 |
| *Solea solea* | Common sole (Solea vulgaris) | 0.0038 | 3.2751 |
| *Soleidae* | Soles (family) | 0.022 | 3.0848 |
| *Somniosus microcephalus* | Greenland shark | 0.0161 | 2.93 |
| *Sparidae* | Porgies (family) | 0.1295 | 2.838 |
| *Spondyliosoma cantharus* | Black seabream | 0.0151 | 3.0233 |
| *Squalidae* | Dogfish sharks (family) | 0.0034 | 3.0955 |
| *Squalus acanthias* | Piked/spiny dogfish, spurdog | 0.0034 | 3.0955 |
| *Stichaeidae* | Pricklebacks (family) | 0.0244 | 2.0439 |
| *Syngnathidae* | Pipefish and seahorses (family) | 0.0002 | 3.2326 |
| *Syngnathus* | Seaweed pipefishes (genus) | 0.0001 | 3.3877 |
| *Syngnathus acus* | Greater pipefish | 0.0001 | 3.527 |
| *Syngnathus rostellatus* | Nilsson's pipefish, lesser pipefish | 0.0001 | 3.414 |
| *Syngnathus typhle* | Broadnosed pipefish | 0.0001 | 3.222 |
| *Taurulus bubalis* | Longspined bullhead | 0.0154 | 3 |
| *Trachinus draco* | Greater weever | 0.0018 | 3.4099 |
| *Trachyrincus murrayi* | Roughnose grenadier (Triglops murrayi) | 0.0088 | 3 |
| *Triglidae* | Searobins (family) | 0.0052 | 3.1457 |
| *Trigloporus lastoviza* | Streaked gurnard | 0.0049 | 3.039 |
| *Triglops murrayi* | Moustache sculpin | 0.0088 | 3 |
| *Triglops pingelii* | Ribbed sculpin | 0.0031 | 3.181 |
| *Triglopsis quadricornis* | Fourhorn sculpin | 0.0031 | 3.181 |
| *Trisopterus esmarkii* | Norway pout | 0.0046 | 3.1405 |
| *Trisopterus luscus* | Bib, pouting | 0.0038 | 3.3665 |
| *Trisopterus minutus* | Poor cod | 0.0092 | 3.0265 |
| *Zeugopterus* | Turbots (genus) | 0.0139 | 3.1457 |
| *Zeugopterus punctatus* | Topknot | 0.0139 | 3.1457 |
| *Zeugopterus regius* | Eckström's topknot | 0.0139 | 3.1457 |
| *Zoarces viviparus* | Eelpout | 0.0417 | 2.2532 |
| *Zoarcidae* | Eelpouts (family) | 0.0417 | 2.2532 |

**Table S1**. Species used in analysis, all from the North Sea IBTS quarter 1 trawl, 1980-2008 with Latin name (column 1), common name (column 2), conversion factors a and b for length to weight conversion (columns 3 and 4 respectively). Latin names with another Latin names in brackets are alternative names given to the same species.

**Table S2**

| *Year* | *Cod* | *Haddock* | *Norway Pout* | *Plaice* | *Saithe* | *Sole* | *Whiting* |
| --- | --- | --- | --- | --- | --- | --- | --- |
| 1980 | 0.86 | 0.899 | **0.601** | 0.555 | 0.443 | 0.452 | **0.518** |
| 1981 | 0.89 | 0.659 | **0.352** | 0.538 | 0.306 | 0.497 | **0.401** |
| 1982 | 0.983 | 0.659 | **0.648** | 0.602 | 0.469 | 0.543 | **0.401** |
| 1983 | 0.972 | 0.884 | 0.901 | 0.594 | 0.548 | 0.488 | **0.510** |
| 1984 | 0.916 | 0.873 | 1.284 | 0.585 | 0.677 | 0.618 | **0.505** |
| 1985 | 0.887 | 0.872 | 1.334 | 0.531 | 0.714 | 0.599 | **0.504** |
| 1986 | 0.936 | 1.203 | 1.112 | 0.662 | 0.819 | 0.58 | **0.665** |
| 1987 | 0.938 | 1.024 | 0.906 | 0.692 | 0.645 | 0.491 | **0.578** |
| 1988 | 0.948 | 1.108 | 0.661 | 0.666 | 0.621 | 0.566 | **0.619** |
| 1989 | 0.966 | 0.952 | 0.833 | 0.61 | 0.673 | 0.439 | **0.543** |
| 1990 | 0.906 | 1.114 | 0.76 | 0.567 | 0.598 | 0.44 | 0.6586 |
| 1991 | 0.91 | 0.888 | 0.894 | 0.649 | 0.576 | 0.445 | 0.5222 |
| 1992 | 0.877 | 0.98 | 0.934 | 0.631 | 0.63 | 0.421 | 0.4852 |
| 1993 | 0.891 | 0.896 | 0.842 | 0.637 | 0.529 | 0.508 | 0.4878 |
| 1994 | 0.906 | 0.83 | 1.074 | 0.619 | 0.509 | 0.567 | 0.624 |
| 1995 | 0.934 | 0.733 | 0.587 | 0.643 | 0.411 | 0.538 | 0.5198 |
| 1996 | 0.955 | 0.688 | 0.448 | 0.673 | 0.401 | 0.706 | 0.4172 |
| 1997 | 0.961 | 0.537 | 0.59 | 0.796 | 0.283 | 0.608 | 0.327 |
| 1998 | 0.98 | 0.604 | 0.299 | 0.735 | 0.34 | 0.646 | 0.312 |
| 1999 | 0.999 | 0.714 | 0.651 | 0.666 | 0.349 | 0.579 | 0.3764 |
| 2000 | 0.995 | 0.765 | 0.585 | 0.475 | 0.299 | 0.608 | 0.4358 |
| 2001 | 0.956 | 0.492 | 0.266 | 0.77 | 0.273 | 0.581 | 0.308 |
| 2002 | 0.926 | 0.229 | 0.512 | 0.58 | 0.244 | 0.578 | 0.2064 |
| 2003 | 0.901 | 0.201 | 0.248 | 0.61 | 0.224 | 0.59 | 0.1868 |
| 2004 | 0.857 | 0.263 | 0.156 | 0.481 | 0.189 | 0.514 | 0.1916 |
| 2005 | 0.8 | 0.31 | 0 | 0.41 | 0.25 | 0.584 | 0.1834 |
| 2006 | 0.723 | 0.511 | 0.286 | 0.375 | 0.266 | 0.455 | 0.2708 |
| 2007 | 0.669 | 0.398 | 0.025 | 0.317 | 0.252 | 0.466 | 0.2606 |
| 2008 | 0.63 | 0.227 | 0.137 | 0.237 | 0.344 | 0.369 | 0.2664 |
| 2009 | 0.602 | 0.209 | 0.250 | 0.211 | 0.388 | 0.364 | 0.229 |
| 2010 | 0.583 | 0.233 | 0.421 | 0.206 | 0.289 | 0.355 | 0.2278 |
| 2011 | 0.572 | 0.298 | 0.034 | 0.229 | 0.284 | 0.296 | 0.1742 |

**Table S2.** Fishing North Sea mortalities for each species from 1980 – 2011. All data is from ICES except in values in bold: Norway pout values calculated from saithe (r^2^ = 0.74), whiting from haddock values (r^2^ = 0.87).

**Table S3.**

|  | 1977 | 1978 | 1979 | 1980 | 1981 | 1982 | 1983 | 1984 | 1985 | 1986 | 1987 |
| --- | --- | --- | --- | --- | --- | --- | --- | --- | --- | --- | --- |
| Belgium | 11030 | 10797.5 | 12075.5 | 10075.5 | 9916.25 | 11742 | 14811 | 17168 | 16655.25 | 11745.25 | 12707.75 |
| Denmark | 23604 | 24435 | 31930 | 31917 | 28839 | 30911 | 28301 | 31501 | 35980 | 31510 | 27754.25 |
| Faeroe Islands | 105 | 30 | 0 | 189 | 23 | 3 | 16 | 0 | 15 | 59 | 41 |
| France | 1944.5 | 2332.5 | 2701.25 | 2791.25 | 4543 | 3347.25 | 3199.25 | 3653 | 3369 | 2175 | 3431 |
| Germany | 0 | 0 | 0 | 0 | 0 | 0 | 0 | 0 | 0 | 0 | 0 |
| Germany Fed Rep | 6537.5 | 5856.75 | 5185.25 | 5234.5 | 4215.5 | 4311 | 3502.5 | 3916 | 3053.25 | 2484.5 | 2718.75 |
| Greenland | 0 | 0 | 0 | 0 | 0 | 0 | 0 | 0 | 0 | 0 | 0 |
| Ireland | 22 | 37.75 | 63 | 0 | 5.5 | 0 | 0 | 0 | 0 | 0 | 0 |
| Netherlands | 63825 | 45083.25 | 58487 | 63238 | 62657 | 88623.25 | 86769.75 | 75886 | 105933 | 84094 | 87247 |
| Norway | 936.5 | 755 | 1195.75 | 1251.75 | 1060 | 897.75 | 971.75 | 999.5 | 1085.75 | 671.5 | 896.5 |
| Poland | 1 | 0 | 0 | 0 | 0 | 0 | 0 | 0 | 0 | 0 | 0 |
| Portugal | 0 | 0 | 0 | 0 | 0 | 0 | 0 | 0 | 0 | 0 | 0 |
| Spain | 0 | 0 | 0 | 0 | 0 | 0 | 0 | 0 | 0 | 0 | 0 |
| Sweden | 0 | 0 | 14 | 12 | 4 | 14.25 | 30.25 | 23 | 26 | 24 | 16.25 |
| UK - Eng + Wales + NI | 0 | 0 | 0 | 0 | 0 | 0 | 0 | 0 | 0 | 0 | 0 |
| UK - Eng + Wales | 33598.75 | 34305.75 | 32195.5 | 24504.75 | 22048.25 | 21861.5 | 18358.5 | 18328.75 | 16786.25 | 17254.25 | 19899 |
| UK - NI | 0 | 0 | 0 | 0 | 0 | 0 | 0 | 0 | 0 | 0 | 0 |
| UK - Scotland | 11783 | 12403.75 | 12152.5 | 13349.25 | 11495.25 | 11444.5 | 12957.25 | 14778.75 | 16733.75 | 17053.25 | 21471 |
| USSR | 9 | 0 | 0 | 0 | 0 | 0 | 0 | 0 | 0 | 0 | 0 |

|  | 1988 | | 1989 | | 1990 | | 1991 | | 1992 | | 1993 | | 1994 | | 1995 | | 1996 | | 1997 | | 1998 | |
| --- | --- | --- | --- | --- | --- | --- | --- | --- | --- | --- | --- | --- | --- | --- | --- | --- | --- | --- | --- | --- | --- | --- |
| Belgium | 15466 | | 14632 | | 18628 | | 20231 | | 17055 | | 17021 | | 13870 | | 12730 | | 11142 | | 8953 | | 10418 | |
| Denmark | 25985.25 | | 29493 | | 33550.25 | | 31839.5 | | 27782.5 | | 24583.5 | | 24423.75 | | 19850.75 | | 18955 | | 20104.25 | | 15054 | |
| Faeroe Islands | 118 | | 22 | | 17 | | 49 | | 17 | | 13 | | 124 | | 732 | | 0 | | 16 | | 13 | |
| France | 3494 | | 2961 | | 2447 | | 2064 | | 2364 | | 2090 | | 1928 | | 2179 | | 2229 | | 1116 | | 1551 | |
| Germany | 0 | | 0 | | 0 | | 12480.75 | | 10169 | | 9649.75 | | 9487.75 | | 10628.25 | | 7035.25 | | 6922.25 | | 5818 | |
| Germany Fed Rep | | 3848 | | 6913.5 | | 12272.5 | | 0 | | 0 | | 0 | | 0 | | 0 | | 0 | | 0 | |  |
| Greenland | 0 | | 0 | | 0 | | 0 | | 0 | | 0 | | 0 | | 0 | | 0 | | 0 | | 0 | |
| Ireland | 0 | | 0 | | 0 | | 0 | | 0 | | 0 | | 0 | | 0 | | 0 | | 0 | | 10 | |
| Netherlands | 95342.25 | | 102226 | | 100939 | | 91938 | | 74801 | | 75756 | | 77734 | | 69010 | | 53484 | | 47113 | | 62809 | |
| Norway | 717 | | 1376 | | 2903.75 | | 2190.5 | | 3297.75 | | 3977.5 | | 3406.75 | | 2253 | | 3769.5 | | 3636.25 | | 3237.25 | |
| Poland | 0 | | 0 | | 0 | | 0 | | 0 | | 0 | | 0 | | 0 | | 0 | | 0 | | 0 | |
| Portugal | 0 | | 0 | | 0 | | 0 | | 0 | | 0 | | 0 | | 0 | | 0 | | 0 | | 0 | |
| Spain | 0 | | 0 | | 0 | | 0 | | 0 | | 0 | | 0 | | 0 | | 0 | | 0 | | 0 | |
| Sweden | 9 | | 29 | | 250 | | 219 | | 119 | | 67 | | 24.75 | | 20 | | 12.75 | | 27.75 | | 24.75 | |
| UK - Eng + Wales + NI | 0 | | 25747.75 | | 24993.75 | | 24839.5 | | 28047.5 | | 27947 | | 25430.25 | | 23354 | | 20255.75 | | 21651.75 | | 17309.25 | |
| UK - Eng + Wales | 22622 | | 0 | | 0 | | 0 | | 0 | | 0 | | 0 | | 0 | | 0 | | 0 | | 0 | |
| UK - NI | 0.5 | | 0 | | 0 | | 0 | | 0 | | 0 | | 0 | | 0 | | 0 | | 0 | | 0 | |
| UK - Scotland | 22233.75 | | 20478.75 | | 22117.25 | | 26221.5 | | 27184.75 | | 28940.25 | | 30994 | | 33622 | | 40191.5 | | 37668.75 | | 31842.5 | |
| USSR | 0 | | 0 | | 0 | | 0 | | 0 | | 0 | | 0 | | 0 | | 0 | | 0 | | 0 | |

|  | 1999 | 2000 | 2001 | 2002 | 2003 | 2004 | 2005 | 2006 | 2007 | 2008 |
| --- | --- | --- | --- | --- | --- | --- | --- | --- | --- | --- |
| Belgium | 10882.5 | 12039.25 | 11236.5 | 8852 | 8220.5 | 8166 | 6846 | 6351 | 6891 | 6817.25 |
| Denmark | 19127.75 | 19224.25 | 19801.5 | 18325.5 | 20297.75 | 18442.75 | 17575 | 17374 | 12658.25 | 12934.5 |
| Faeroe Islands | 8 | 0 | 2 | 12 | 3 | 11 | 22 | 2 | 1 | 0 |
| France | 0 | 1875 | 1858.5 | 1773.5 | 1570.5 | 1277 | 1221 | 1206 | 1280.5 | 1108.25 |
| Germany | 6290 | 7343 | 7208 | 5961 | 5877 | 5987 | 6070 | 6237.25 | 4345 | 4851 |
| Germany Fed Rep | 0 | 0 | 0 | 0 | 0 | 0 | 0 | 0 | 0 | 0 |
| Greenland | 0 | 0 | 0 | 0 | 0 | 0 | 0 | 0 | 0 | 8 |
| Ireland | 21 | 19 | 13 | 3 | 2 | 0.25 | 0 | 0 | 0 | 0 |
| Netherlands | 69577 | 64075 | 59752 | 53766 | 52090 | 48749 | 45551 | 43829 | 45373 | 40994 |
| Norway | 4602.5 | 3224.5 | 4028 | 3524.5 | 3498.5 | 3837.5 | 3253 | 2956.25 | 2553 | 2295 |
| Poland | 0 | 0 | 0 | 0 | 0 | 0 | 0 | 0 | 0 | 0 |
| Portugal | 0 | 0 | 0 | 0 | 0 | 0 | 0 | 3 | 0 | 0 |
| Spain | 0 | 0 | 0 | 0 | 0 | 0 | 0 | 0 | 0 | 0 |
| Sweden | 26 | 99.5 | 69 | 80.25 | 21 | 15 | 16 | 14.5 | 45 | 123 |
| UK - Eng + Wales + NI | 14798.75 | 17304.75 | 15102 | 11425 | 10066.75 | 10298 | 9122 | 9250.25 | 9290.5 | 8656.5 |
| UK - Eng + Wales | 0 | 0 | 0 | 0 | 0 | 0 | 0 | 0 | 0 | 0 |
| UK - NI | 0 | 0 | 0 | 0 | 0 | 0 | 0 | 0 | 0 | 0 |
| UK - Scotland | 26639 | 25656.25 | 26110 | 23195.25 | 18177.25 | 18813.75 | 13681 | 18626.25 | 19114.75 | 18640 |
| USSR | 0 | 0 | 0 | 0 | 0 | 0 | 0 | 0 | 0 | 0 |

**Table S3**. Beam landings data for all countries that fish in the North Sea 1977 – 2008 for all expected beam-related catches.

**Table S4.**

|  | 1977 | 1978 | 1979 | 1980 | 1981 | 1982 | 1983 | 1984 | 1985 |
| --- | --- | --- | --- | --- | --- | --- | --- | --- | --- |
| Belgium | 18029 | 25026.5 | 19235.25 | 15871.5 | 13999.25 | 11273.25 | 12526 | 11052 | 8845.75 |
| Denmark | 384794 | 255798 | 332549 | 471872 | 321051 | 408000 | 383794.5 | 300256.25 | 273889.5 |
| Faeroe Islands | 50511 | 18889 | 23309 | 35646 | 17232 | 13407 | 32013 | 19067 | 10953 |
| France | 78197.5 | 85447.75 | 94910 | 85681.5 | 100446.25 | 100302.5 | 93699 | 88399 | 69490 |
| Germany | 0 | 0 | 0 | 0 | 0 | 0 | 0 | 0 | 0 |
| Germany Fed Rep | 55920.25 | 69730.5 | 44539.75 | 42109.25 | 42692 | 37642.25 | 39050.5 | 42643 | 33952.5 |
| Germany New | 2465 | 2583 | 1659 | 1024 | 0 | 0 | 0 | 0 | 0 |
| Greenland | 0 | 0 | 0 | 0 | 0 | 0 | 0 | 0 | 0 |
| Iceland | 0 | 0 | 0 | 0 | 0 | 0 | 0 | 0 | 0 |
| Ireland | 1251 | 534 | 1 | 0 | 0 | 0 | 0 | 0 | 0 |
| Lithuania | 0 | 0 | 0 | 0 | 0 | 0 | 0 | 0 | 0 |
| Netherlands | 50832 | 68006.25 | 52369.5 | 63314.75 | 70842.25 | 52726.25 | 50378.25 | 35644 | 42115 |
| Norway | 175873.25 | 183531.75 | 173702 | 198967.25 | 167635.75 | 276992.5 | 298220.5 | 296409.25 | 245674.25 |
| Poland | 14039 | 5918 | 6392 | 2536 | 737 | 1219 | 642 | 441 | 0 |
| Portugal | 0 | 0 | 0 | 0 | 0 | 0 | 0 | 0 | 0 |
| Russian Fed | 0 | 0 | 0 | 0 | 0 | 0 | 0 | 0 | 0 |
| Spain | 0 | 0 | 0 | 0 | 0 | 0 | 0 | 0 | 0 |
| Sweden | 3820 | 0 | 1838.25 | 1880 | 1950.25 | 2696 | 2456 | 2851 | 4219 |
| UK - Eng + Wales + NI | 0 | 0 | 0 | 0 | 0 | 0 | 0 | 0 | 0 |
| UK - Eng + Wales | 75702.25 | 99016.5 | 89898.75 | 83159 | 91924.5 | 86526.5 | 85238.75 | 65368.75 | 58037 |
| UK - NI | 0 | 0 | 0 | 0 | 0 | 0 | 0 | 0 | 0 |
| UK - Scotland | 181110 | 170675 | 160889.75 | 163996 | 179518 | 206614 | 211159 | 197012.75 | 220475 |
| USSR | 56810 | 10232 | 2082 | 0 | 0 | 0 | 0 | 0 | 0 |

|  | 1986 | 1987 | 1988 | 1989 | 1990 | 1991 | 1992 | 1993 | 1994 |
| --- | --- | --- | --- | --- | --- | --- | --- | --- | --- |
| Belgium | 10192.25 | 9183 | 8775.5 | 5847 | 5636 | 4880 | 6328 | 6333 | 5597 |
| Denmark | 228509.25 | 236789 | 231337 | 219597.5 | 140382 | 156288.25 | 215644.25 | 179780.25 | 158474.25 |
| Faeroe Islands | 2735 | 5623 | 2032 | 1566 | 2665 | 2152 | 5232 | 5325 | 5541 |
| France | 69114 | 65764 | 54230 | 43449 | 41082 | 25158 | 21266 | 26643 | 23582 |
| Germany | 0 | 0 | 0 | 0 | 0 | 29226 | 24226 | 23988 | 19738 |
| Germany Fed Rep | 33054.5 | 32737.75 | 28265.75 | 27841.25 | 29387.25 | 0 | 0 | 0 | 0 |
| Germany New | 0 | 0 | 0 | 0 | 0 | 0 | 0 | 0 | 0 |
| Greenland | 0 | 0 | 0 | 0 | 0 | 0 | 0 | 0 | 0 |
| Iceland | 0 | 0 | 0 | 0 | 0 | 0 | 0 | 0 | 0 |
| Ireland | 0 | 0 | 0 | 0 | 0 | 0 | 0 | 0 | 0 |
| Lithuania | 0 | 0 | 0 | 0 | 0 | 91 | 0 | 0 | 0 |
| Netherlands | 40776 | 31416 | 23718.75 | 17670 | 12357 | 11500 | 17308 | 15565 | 10699 |
| Norway | 161089.75 | 165975.5 | 113173.75 | 169317.5 | 168387 | 172063 | 230268.25 | 168341.75 | 150747.75 |
| Poland | 506 | 868 | 1069 | 901 | 1350 | 1370 | 1311 | 1022 | 194 |
| Portugal | 0 | 0 | 0 | 0 | 0 | 0 | 0 | 0 | 0 |
| Russian Fed | 0 | 0 | 0 | 0 | 0 | 0 | 0 | 0 | 0 |
| Spain | 0 | 0 | 0 | 0 | 0 | 0 | 0 | 0 | 0 |
| Sweden | 3936 | 3071.25 | 2153.5 | 2307 | 2617 | 4542 | 4143 | 3732 | 2472.25 |
| UK - Eng + Wales + NI | 0 | 0 | 0 | 31279.75 | 28243.25 | 26705 | 26537 | 27413.25 | 25705.5 |
| UK - Eng + Wales | 45274.75 | 48731 | 40827 | 0 | 0 | 0 | 0 | 0 | 0 |
| UK - NI | 0 | 0 | 6.25 | 0 | 0 | 0 | 0 | 0 | 0 |
| UK - Scotland | 223385 | 191812.5 | 176619.25 | 129294.5 | 108196 | 113708.25 | 119017.5 | 143372 | 146471.25 |
| USSR | 0 | 0 | 0 | 0 | 0 | 116 | 0 | 0 | 0 |

|  | 1995 | 1996 | 1997 | 1998 | 1999 | 2000 | 2001 | 2002 |
| --- | --- | --- | --- | --- | --- | --- | --- | --- |
| Belgium | 7596 | 6164 | 7073 | 8139 | 6034.5 | 5433.5 | 4467 | 4431.5 |
| Denmark | 203723 | 130116.75 | 138576 | 75581 | 72889.25 | 154170 | 61651.5 | 89168.5 |
| Faeroe Islands | 13134 | 8502 | 7263 | 6197 | 3815 | 0 | 353 | 4392 |
| France | 23844 | 21936 | 20393 | 18995 | 0 | 28791.25 | 29629.25 | 33022.75 |
| Germany | 24225 | 22869.75 | 20335 | 20515 | 15239 | 12290 | 12794 | 14779 |
| Germany Fed Rep | 0 | 0 | 0 | 0 | 0 | 0 | 0 | 0 |
| Germany New | 0 | 0 | 0 | 0 | 0 | 0 | 0 | 0 |
| Greenland | 0 | 0 | 0 | 0 | 0 | 601 | 1526 | 0 |
| Iceland | 0 | 0 | 0 | 0 | 0 | 0 | 0 | 0 |
| Ireland | 0 | 0 | 0 | 7 | 41 | 33 | 29 | 15 |
| Lithuania | 0 | 0 | 0 | 0 | 0 | 0 | 0 | 0 |
| Netherlands | 15329 | 12940 | 15119 | 17010 | 11148 | 11245 | 9546 | 10528 |
| Norway | 185736.25 | 165899.5 | 102672.5 | 95209.25 | 122230.5 | 111944.75 | 76995.75 | 101101.25 |
| Poland | 645 | 416 | 893 | 869 | 929 | 794 | 781 | 843 |
| Portugal | 0 | 0 | 0 | 0 | 0 | 0 | 0 | 0 |
| Russian Fed | 0 | 0 | 0 | 0 | 0 | 369 | 34 | 0 |
| Spain | 0 | 0 | 0 | 0 | 0 | 0 | 0 | 0 |
| Sweden | 3245 | 2495 | 2881 | 2706.25 | 3330 | 2877.5 | 3035.5 | 2681.25 |
| UK - Eng + Wales + NI | 26169.75 | 26208.5 | 23864.5 | 27704 | 19504 | 12656.75 | 10878 | 11549.5 |
| UK - Eng + Wales | 0 | 0 | 0 | 0 | 0 | 0 | 0 | 0 |
| UK - NI | 0 | 0 | 0 | 0 | 0 | 0 | 0 | 0 |
| UK - Scotland | 147888.5 | 139433.75 | 134103.25 | 129446.5 | 107333 | 89046.75 | 67823 | 76696.75 |
| USSR | 0 | 0 | 0 | 0 | 0 | 0 | 0 | 0 |

|  | 2003 | 2004 | 2005 | 2006 | 2007 | 2008 |
| --- | --- | --- | --- | --- | --- | --- |
| Belgium | 2752.5 | 2903.5 | 2708 | 2082 | 1771.25 | 1688.5 |
| Denmark | 26285.25 | 26445.25 | 14051 | 51918.25 | 8902 | 43306.25 |
| Faeroe Islands | 2892 | 1754 | 582 | 73.5 | 16.25 | 198 |
| France | 23677 | 17474.5 | 15333 | 21296 | 21588.75 | 21681.25 |
| Germany | 13370 | 13365 | 16187 | 17949 | 13872 | 14907 |
| Germany Fed Rep | 0 | 0 | 0 | 0 | 0 | 0 |
| Germany New | 0 | 0 | 0 | 0 | 0 | 0 |
| Greenland | 0 | 0 | 1103 | 969 | 594 | 911 |
| Iceland | 0 | 0 | 0 | 0 | 0 | 0 |
| Ireland | 2 | 1 | 0 | 0 | 0 | 0 |
| Lithuania | 0 | 0 | 149 | 0 | 0 | 0 |
| Netherlands | 6436 | 5645 | 5421 | 5108 | 5161 | 5270 |
| Norway | 87084.25 | 80614 | 80449 | 88322 | 66886.25 | 82942.75 |
| Poland | 812 | 0 | 1100 | 1092 | 1394 | 1425 |
| Portugal | 0 | 0 | 0 | 1053 | 0 | 0 |
| Russian Fed | 0 | 0 | 35 | 2 | 5 | 5 |
| Spain | 0 | 0 | 2 | 0 | 0 | 0 |
| Sweden | 2025.5 | 2141.25 | 2074 | 1455.5 | 1433 | 1791 |
| UK - Eng + Wales + NI | 6465.5 | 5273.75 | 6096 | 7469 | 9947.5 | 6753.25 |
| UK - Eng + Wales | 0 | 0 | 0 | 0 | 0 | 0 |
| UK - NI | 0 | 0 | 0 | 0 | 0 | 0 |
| UK - Scotland | 57032.5 | 61959.75 | 43067 | 58360.25 | 50735 | 58293.25 |
| USSR | 0 | 0 | 0 | 0 | 0 | 0 |

**Table S4**. Otter landings data for all countries that fish in the North Sea from 1977 – 2008 based on expected otter-related landings.

**References**

Baretta JW, Ebenhöh W, Ruardij P (1995) The European regional seas ecosystem model, a complex marine ecosystem model. *Netherlands Journal of Sea Research*, **33**, 233–246.

Burchard H, Bolding K (2002) *GETM - a general estuarine transport mode. Scientific Documentation.* European Commission.

Burchard H, Bolding K, Umlauf L (2014) *GETM Source Code and Test Case Documentation*.

Cefas (2014) *Trawling through time:Cefas science and data 1902-2014*. Cefas, Lowestoft, 16pp.

Ebenhöh W, Baretta-Bekker JG, Baretta JW (1997) The primary production module in the marine ecosystem model ERSEM II, with emphasis on the light forcing, *Journal of Sea Research*, **38**, pp 173–193

Engelhard GH, Lynam CL, García-Carreras B, Dolder PJ, Mackinson S (2015) Effort reduction and the large fish indicator: spatial trends reveal positive impacts of recent European fleet reduction schemes. *Environmental Conservation*, **42**. In press.

Jennings S, Alvsvag J, Cotter AJR et al. (1999) Fishing effects in northeast Atlantic shelf seas: patterns in fishing effort, diversity and community structure. III. International trawling effort in the North Sea: an analysis of spatial and temporal trends. *Fisheries Research*, **40**, 125–134.

Daan N (2001) *The IBTS dataset: a plea for quality control*. ICES CM 2001/T:03.

Daan N (1997) TAC management of North Sea flatfish fisheries. *Journal of Sea Research*, **37**, 321–340*.*

Fung T, Farnsworth KD, Reid DG, Rossberg AG (2012) Recent data suggest no further recovery in North Sea Large Fish Indicator. *ICES Journal of Marine Science*, **69**, 235–239.

Ruardij P, Raaphorst W (1995) Benthic nutrient regeneration in the ERSEM-BFM ecosystem model of the North Sea, *Journal of Sea Research*, **33**, pp 453–483, doi:10.1016/0077-7579(95)90057-8

Ruardij P, Van Haren H, Ridderinkhof H (1997) The impacts of thermal stratification on phytoplankton and nutrient dynamics in shelf seas: a model study, *Journal of Sea Research*., **38**, pp 311–331, doi:10.1016/S1385-1101(97)00042-7

Ruardij P, Veldhuis MJW, Brussard CPD (2005) Modeling the bloom dynamics of the polymorphic phytoplankter Phaeocystis globosa: impact of grazers and viruses, *Harmful Algae*, **4**, pp 941–963, doi:10.1016/j.hal.2004.12.011

Stips A, Bolding K, Pohlmann T, Burchard H (2004) Stimulating the temporal and spatial dynamics of the North Sea using the new model GETM (general estuarine transport model). *Ocean Dynamics*, **54**, 266–283.

Van der Molen J, Aldridge JN, Coughlan C, Parker ER, Stephens D, Ruardij P (2013) Modelling marine ecosystem response to climate change and trawling in the North Sea, *Biogeochemistry*, **113**, 1–24, doi:10.1007/s10533-012-9763-7

Van der Molen J, Smith HCM, Lepper P, Limpenney S, Rees J (2014) Predicting the largescale consequences offshore wind turbine array development on a North Sea ecosystem, *Continental Shelf Research*, **85**, 60–72, doi:10.1016/j.csr.2014.05.018

Van Leeuwen SM, van der Molen J, Ruardij P, Fernand L, Jickells T (2013) Modelling the contribution of Deep Chlorophyll Maxima to annual primary production in the North Sea, *Biogeochemist*ry, **113,** 137–152, doi:10.1007/s10533-012-9704-5

Van Leeuwen SM, Tett P, Mills DK, van der Molen J (2015) Stratified and non-stratified areas in the North Sea: long-term variability and biological and policy implications, *Journal of Geophysical Research-Oceans*, **120 (7)**, pp 4670 – 4686, doi:10.1002/2014JC010485

Vichi M, May W, Navarra, A.(2003) Response of a complex ecosystem model of the northern Adriatic Sea to a regional climate change scenario, *Climate Research*, **24**, 141–158, doi:10.3354/cr024141

Vichi M, Ruardij P, Baretta JW (2004) Link or sink: a modelling interpretation of the open Baltic biogeochemistry, *Biogeosciences*, **1**, 79–100, doi:10.5194/bg-1-79-2004

Vichi M, Pinardi N, Masina, S (2007) A generalized model of pelagic biogeochemistry for the global ocean ecosystem. Part I: Theory, *Journal of Marine Systems*, **64**, pp 89–109,doi:10.1016/j.jmarsys.2006.03.006

Wei HL, Billings SA, Liu J (2004) Term and variable selection for nonlinear system indentification. *International Journal of Control*, **77**, 86-110.

Wei HL, Billings SA (2008) Generalized cellular neural networks (GCNNs) constructed using particle swarm optimization for spatio-temporal evolutionary pattern identification. *International Journal of Bifurcation and Chaos*, **18(12)**, 3611–3624.

Wei HL, Billings SA, Zhao Y, Guo LZ (2010) An adaptive wavelet neural network for spatio-temporal system identification. *Neural Networks*, **23**, 1286-1299.

WGMIXFISH (2014) ICES Working Group on Mixed Fisheries, *North Sea Report*, Executive summary.
